# A bibliometric study of the most-cited research articles and reviews in Naunyn–Schmiedeberg’s Archives of Pharmacology (1969–2024)

**DOI:** 10.1007/s00210-025-04471-7

**Published:** 2025-08-01

**Authors:** Roland Seifert, Waseem Hassan

**Affiliations:** 1 Institute of Pharmacology, Hannover Medical School, Carl-Neuberg-Str. 1, Hannover, D-30625 Germany; 2 Institute of Chemical Sciences, University of Peshawar, Peshawar, 25120 Khyber Pakhtunkhwa Pakistan

**Keywords:** NSAP, Most-cited research articles, Most-cited reviews, Top contributors, Co-words analysis, Editorial policies

## Abstract

The objective of this study was to perform a comprehensive bibliometric analysis of the top 100 most-cited research articles and the top 100 most-cited reviews published in Naunyn–Schmiedeberg’s Archives of Pharmacology (NSAP) between 1969 and 2024. The Scopus database was systematically analyzed for research papers and reviews. Author performance for both research article and review groups was evaluated using multiple bibliometric indicators—total publications, citations, h-index, g-index, m-index, HG composite, and Q2 index—with detailed results presented in the supplementary files. Institutional contributions were also highlighted. Furthermore, co-word analysis of research article and review titles was conducted to identify prevailing thematic focuses. During this period, the journal published a total of 12,354 research articles, with publication trends showing notable fluctuations. A record high of 706 research articles was published in 2024. Germany led overall contributions with 4852 publications, followed by the USA (860), Japan (661), and China (507), with the latter demonstrating a steep rise in recent years. The top 100 most-cited research articles, published between 1970 and 2006, garnered an average of 262.5 citations each. Peak citation averages occurred in 1984 (456.67), 1979 (392.20), and 1986 (384.00), while the highest annual citation rates were recorded in 2006 (15.20), 2004 (10.91), and 1984 (10.87). These influential research articles, many of them in the field of neurotransmitter research, involved 314 authors, averaging 3.83 co-authors per paper, with 15% reflecting international collaborations. Germany (32), Sweden (16), and Switzerland (12) emerged as the leading contributing countries. Similarly, review publications demonstrated a steady increase over time, culminating in a sharp rise in 2024 with 227 reviews—out of a total of 560. India (142) and Germany (140) were the most prolific countries in review authorship, followed by the USA and Iran. The top 100 most-cited reviews, published from 1973 to 2023, averaged 132.2 citations per research article. Citation activity peaked in the early 2000 s, with a 2002 review receiving 545 citations and the year 2000 averaging 277.8 citations per review. Overall, NSAP has made substantial contributions in specific areas of research and more broadly with respect to reviews. Very substantial changes in geographic origin of papers occurred during the past two decades, reflecting internationalization. Our analysis provides the scientific basis for recently implemented “Collections and call for papers” on diverse topics and editorial policies regarding review papers. Thus, the bibliographic analysis of a journal can be effectively used as a tool to ensure its future existence and adapt to a changing scientific environment.

**Supplementary Information:**

The online version contains supplementary material available at 10.1007/s00210-025-04471-7.

## Introduction

Naunyn–Schmiedeberg’s Archives of Pharmacology (NSAP) has been consistently publishing important contributions across neuropharmacology, cardiovascular pharmacology, and exploring drug actions at cellular, biochemical, and molecular levels over the past decades (Dats et al. 2023). Later, the journal has begun to publish papers in the fields of molecular phytopharmacology, immunopharmacology, and pharmacology of malignant diseases (Dats et al. 2023). Recently, the journal has further expanded its scope by introducing thematic collections in various novel fields including teaching pharmacology, drug repurposing, probiotics, antibacterial resistance, clinical pharmacology, and pharmacovigilance, bibliometric analysis of pharmacology, pharmacology and society, scientific integrity, drug development and pharmacoeconomics, dietary supplements, history of pharmacology, meeting reports, and news of the German Society for Experimental and Clinical Pharmacology and Toxicology (DGPT) (https://link.springer.com/journal/210/collections?filter=Open).

To mark the journal’s 150th anniversary in 2023, a detailed bibliometric study was conducted by Dats et al. (2023). Research article themes from six representative years (1970, 1980, 1990, 2000, 2010, and 2020) were studied. In addition, geographical trends and the impact factor development were studied to better understand its scholarly influence and international reach. A follow-up study by Basol and Seifert (2024) focused on the journal’s evolution between 1947 and 1974. More recently, our group expanded the bibliometric investigation of NSAP through a series of in-depth studies. Hassan et al. (2025) analyzed 25,931 publications from 1873 to 2025, illustrating a shift from national to global participation, with contributions from 104 countries. While Germany maintained a central role historically, the prominence of countries such as China, India, and Iran has grown markedly in recent years. Abdelwahab et al. (2025a) compared German-authored pharmacology papers in NSAP to those published elsewhere. Between 2019 and 2024, only 192 German papers were published in NSAP, in contrast to 16,775 research articles appearing in other journals, reflecting a trend toward publishing in higher-impact venues. In another study, Abdelwahab et al. (2025b) conducted the first focused bibliometric analysis of research supported by the Deutsche Forschungsgemeinschaft (DFG) in NSAP. Based on Scopus data from 1969 onward, it was found that DFG-sponsored pharmacology research articles in NSAP were relatively rare, averaging fewer than ten publications annually from 2015 to 2023. Furthermore, Abdelwahab et al. (2025c) examined authorship trends in 13,422 NSAP papers published between 1969 and 2024. Using over 250,000 citation records and multiple bibliometric indicators (e.g., h-index, g-index, Q2 index, and HG composite), this study provided a detailed assessment of author productivity and influence over time.

These bibliometric analyses not only reflect the continued evolution of NSAP but also underscore its enduring role in advancing pharmacological research. To extend this line of inquiry and incorporate updated data, the current project focuses on analyzing the top 100 most-cited research articles and top 100 most cited reviews, published between 1969 and 2024. This new analysis seeks to deepen our understanding of the journal’s most influential contributions and its role in shaping the field over the past five decades.

## Materials and methods

This bibliometric analysis was conducted using the Scopus database, selected for its extensive and reliable coverage of peer-reviewed scientific literature. The scope of the analysis was limited to publications from the journal *Naunyn–Schmiedeberg’s Archives of Pharmacology (NSAP)*, covering the period from January 1, 1969, to December 31, 2024. The search was restricted to two document types: research articles and reviews, which were retrieved separately to enable focused analyses within each category.

All bibliographic data—including author names, research article titles, publication years, institutional affiliations, citation counts, and keywords—were exported in CSV format. Duplicate entries and irrelevant document types were removed, and data were verified manually to ensure the accuracy of classification. This preparation allowed for a consistent and structured analysis of publication patterns, citation trends, and scholarly influence within the journal. The analysis was organized into two primary domains.

The first domain focused on research articles, and the second on reviews. Each of these domains was assessed in two phases. In the case of research articles, the first phase included all research articles published during the study period. These were analyzed to determine annual publication trends and major contributors (authors, universities, and countries). The second phase involved isolating the 100 most cited research articles based on citation data indexed in Scopus. These highly cited papers were evaluated separately to gain deeper insights into the most influential contributions, including their citation density (i.e., citations per year), year of publication, and author-specific productivity and impact. Similarly, the reviews were studied in two phases. In the first phase, all published reviews were examined to map trends in publication frequency and top contributors. The second phase extracted the 100 most cited reviews from the dataset, which were subjected to further bibliometric scrutiny. This included calculation of citation density and high-impact contributors within this specific publication type.

For both research articles and reviews, a wide range of bibliometric indicators was used to evaluate performance and influence. These included total number of publications, total citations, the h-index (which reflects both productivity and impact), the g-index (which gives additional weight to highly cited publications), the m-index (which normalizes h-index by scientific age), the HG-index (a harmonic mean of h and g), and the Q2-index (a harmonic mean of h and m). These metrics were used to assess the scholarly contributions of individual authors within the subset of top 100 most cited documents in each category.

Microsoft Excel was used for initial data organization, including sorting, filtering, and computing basic metrics. VOSviewer was utilized for generating network visualizations, allowing for the exploration of co-authorship relationships, institutional collaboration networks, and keyword co-occurrence patterns. In addition, R Studio (using the Bibliometrix and Biblioshiny packages) facilitated more advanced statistical analyses.

This integrated, multi-tool strategy ensured a comprehensive, accurate, and methodologically sound evaluation of *NSAP*’s scholarly contributions over a span of more than five decades.

## Results and discussion

### Part one—analysis of research articles

#### The annual publication details

From 1969 to 2024, a total of 13,267 documents were published in Naunyn–Schmiedeberg’s Archives of Pharmacology. Among these, 12,354 were research articles, while 560 were reviews, which were the focus of this bibliometric analysis. This shows that historically, NSAP has been a journal dedicated to original research. Additionally, the dataset included 163 errata, 90 conference papers, 53 editorials, 36 retracted papers, 8 letters, 2 notes, and 1 short survey. The very low number of retractions demonstrates the high level of scientific integrity of the journal.

For detailed analysis, we focused on research articles and reviews.

A total of 12,354 research articles were published in NSAP from 1969 to 2024. The publication trends showed fluctuations over the years, with notable peaks and declines. The data is presented in Table 1. During the initial years (1969–1973), the number of publications remained relatively moderate, ranging between 157 and 305 research articles per year. A significant increase was observed in 1974, where the number of published research articles surged to 791, marking the highest output in the early decades. However, this peak was followed by a decline, with publications fluctuating between 289 and 492 research articles per year from 1975 to 1981. Another notable rise occurred in 1980, when 719 research articles were published. From 1982 to 1997, the publication rate stabilized, with annual outputs mostly ranging between 161 and 228 research articles. The lowest point within this period was recorded in 1983, with 161 research articles. A gradual decline started in the late 1990 s and early 2000 s, where publications dropped below 200 research articles per year, reaching a minimum of 79 research articles in 2005. This low was due to the fact that in the early 2000 s, many previous major contributors to NSAP had retired, leaving a huge scientific and author gap. As a result, NSAP had to develop a new profile.

**Table 1:** Annual publication trends in *Naunyn–Schmiedeberg’s Archives of Pharmacology* (1969–2024), showing the number of research articles published per year

Year	Number of publications	Year	Number of publications
1969	163	1997	216
1970	280	1998	163
1971	157	1999	160
1972	169	2000	153
1973	305	2001	160
1974	791	2002	141
1975	492	2003	153
1976	289	2004	123
1977	474	2005	79
1978	439	2006	66
1979	381	2007	64
1980	719	2008	83
1981	478	2009	112
1982	191	2010	93
1983	161	2011	97
1984	217	2012	104
1985	214	2013	95
1986	224	2014	113
1987	222	2015	95
1988	227	2016	113
1989	228	2017	114
1990	200	2018	114
1991	206	2019	122
1992	213	2020	204
1993	203	2021	173
1994	190	2022	86
1995	194	2023	217
1996	208	2024	706

**Fig. 1:**
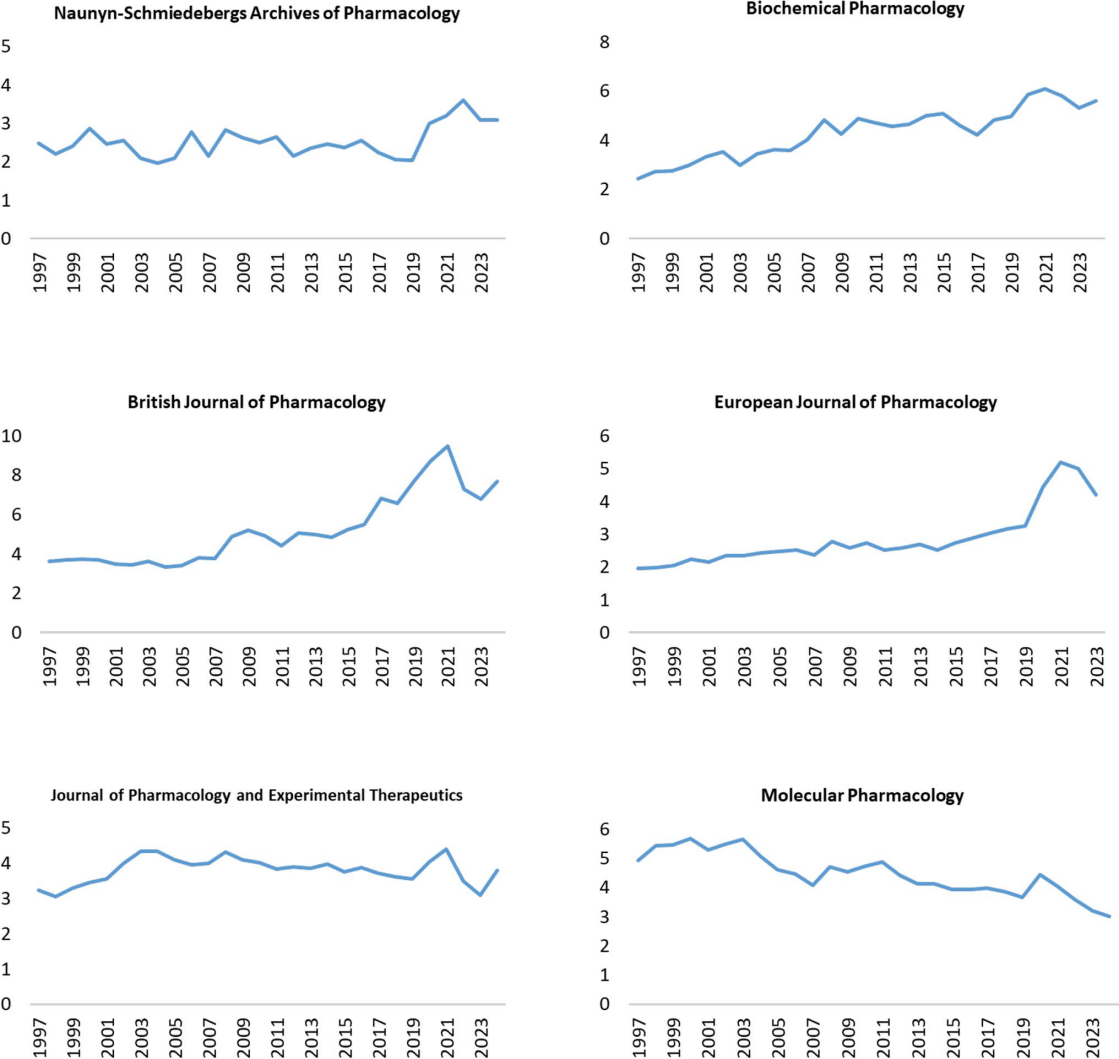
Yearly impact factors (1997–2024) of six leading pharmacology journals: *Naunyn–Schmiedeberg’s Archives of Pharmacology*, *Biochemical Pharmacology*, *British Journal of Pharmacology*, *European Journal of Pharmacology*, *Journal of Pharmacology and Experimental Therapeutics*, and *Molecular Pharmacology*. The data illustrate long-term trends and comparative performance across journals in the pharmacology field

Between 2006 and 2019, the number of published research articles remained relatively low, mostly ranging from 64 to 122 per year. These numbers show how difficult it is for a journal to develop a new scientific focus once a previous research direction was interrupted. However, a sharp increase was observed in 2020, where the number of publications jumped to 204, nearly doubling from the previous years. The trend slightly decreased in 2021, with 173 research articles, before experiencing another decline in 2022, when only 86 research articles were published. A notable recovery was observed in 2023, with 217 research articles, followed by a significant surge in 2024, where 706 research articles were published, marking the highest output in several decades. This sharp increase reflects the successful effort of the journal to integrate novel and diverse research directions into its portfolio (https://link.springer.com/journal/210/collections?filter=Open). Importantly, the increase in research output in NSAP was not accompanied by a decrease in quality, since the Journal Impact Factor (JIF) has remained stable at 3.1 in 2023 and 2024. In fact, the impact factor (IF) data (Fig. 1) over nearly three decades reveal that NSAP has consistently maintained a solid scientific profile among leading pharmacology journals. Although its IF was relatively stable around 2.0–2.8 from 1997 through 2019, a notable upward trend began in 2020, with the journal reaching 3.0 in 2020, peaking at 3.6 in 2022, and maintaining a strong 3.1 in both 2023 and 2024. This recent growth demonstrates the journal’s positive trajectory, rising relevance, and increased recognition in the field.

#### The top contributors

In this part, the details about the top contributors (authors, universities, countries, and sponsors) are provided (Table 2). A threshold of at least 50 published papers was considered to identify the top contributors. Starke, K. emerged as the most influential author, with 142 publications, followed by Göthert, M. with 106 papers. Kaumann, A.J. (82 papers), Trendelenburg, U. (80 papers), and Lembeck, F. (77 papers) were also among the top five contributors. Additionally, Seifert, R. (76 papers), Schlicker, E. (72 papers), Philippu, A. (71 papers), and Habermann, E. (57 papers) maintained a strong research presence. Starke, Göthert, Kaumann, Schlicker, and Philippu terminated their research activities in the early 2000 s, explaining to a substantial extent the decrease in research output of NSAP during this period (supplementary Fig. 1).

**Table 2:** The most prolific contributors to *Naunyn–Schmiedeberg’s Archives of Pharmacology* (1969–2024) research articles, ranked by the total number of published research articles

S#	Author name	At least 50 publications
1	Starke, K	142
2	Göthert, M	106
3	Kaumann, A.J	82
4	Trendelenburg, U	80
5	Lembeck, F	77
6	Seifert, R	76
7	Schlicker, E	72
8	Philippu, A	71
9	Habermann, E	57
10	Hertting, G	56
11	Bönisch, H	55
12	Michel, M.C	51
13	Scholz, H	51
14	Maggi, C.A	50
**S#**	**Affiliation**	**At least 200 publications**
1	Freie Universität Berlin	302
2	Johannes Gutenberg-Universität Mainz	251
3	Universität Freiburg	235
4	Eberhard Karls Universität Tübingen	232
5	Hannover Medical School	211
**S#**	**Country**	**At least 200 publications**
1	Germany	4852
2	USA	860
3	Japan	661
4	China	507
5	UK	492
6	Italy	454
7	Austria	417
8	Switzerland	409
9	Sweden	387
10	France	374
11	Netherlands	323
12	Brazil	260
13	Egypt	250
14	Australia	244
15	India	218
16	Iran	213
17	Poland	212
**S#**	**Funding sponsor**	**At least 100 publications**
1	Deutsche Forschungsgemeinschaft	341
2	National Natural Science Foundation of China	167
3	Conselho Nacional de Desenvolvimento Científico e Tecnológico	160
4	Coordenação de Aperfeiçoamento de Pessoal de Nível Superior	122
5	National Institutes of Health	117

We also explored their publication dynamics of authors over time to identify trends in productivity and career stages (supplementary Fig. 1).

Among the most prolific authors, Starke, K. emerged as a highly active contributor, with a sustained presence from 1969 to 2007. His peak productivity occurred between the mid-1970s and late 1990 s, with the highest output in 1994 (8 papers) and 1995 (9 papers). Göthert, M. followed a similar trajectory, publishing from 1969 to 2010, with a notable increase in activity between 1989 and 1999. His peak year was 1995 (7 papers), and he maintained steady contributions until the early 2000s. Kaumann, A.J. showed moderate but steady productivity from 1972 to 2021. His contributions peaked sporadically, notably in 1980 (5 papers) and 1985 (6 papers), but he remained active even in later years, publishing in 2014, 2016, and 2021. Trendelenburg, U. was highly active in the 1970 s and 1980 s, with a peak in 1974 (9 papers). His contributions declined after 1992. Lembeck, F. also showed strong early contributions, particularly in 1979 (10 papers) and 1980 (8 papers). However, his activity declined significantly after the early 1990s. Schlicker, E. maintained a long career from 1980 to 2022, but his publication output was relatively dispersed. His peak years were in the mid-1990s and 2014 (5 papers). Philippu, A. was most active between the 1970 s and 2000 s, with periodic spikes such as in 1993 (5 papers) and 1999 (6 papers). His return in 2022 suggests continued but reduced involvement in the field. Habermann, E. and Hertting, G. both had strong early contributions, particularly in the 1970s. Habermann, E. peaked in 1973 (8 papers) but had a steep decline after the early 1980s. Hertting, G. had a similar trajectory, with peaks in 1970 (4 papers) and 1980 (6 papers), followed by a gradual decline. Among more recent contributors, Seifert, R. exhibited a significant growth in recent years. Thus, productivity patterns of top contributors are very individual and cannot be categorized easily. This result is consistent with the data on the most highly cited pharmacologists in Germany (Fox and Seifert, 2024).

Institutions with a minimum of 200 publications were identified as key affiliations. Freie Universität Berlin was the leading institution, contributing 302 papers to the journal. Johannes Gutenberg-Universität Mainz followed with 251 publications, while Universität Freiburg (235 papers) and Eberhard Karls Universität Tübingen (232 papers) also had strong research output. Hannover Medical School, with 211 publications, further demonstrated Germany’s leading role in pharmacological research.

Supplementary Fig. 2 depicts the top universities dynamics. Freie Universität Berlin maintained a strong presence in academic research, with a notable peak in the mid-1970s. The institution demonstrated a steady output over several decades. Johannes Gutenberg-Universität Mainz exhibited significant research activity in the 1970 s, with notable contributions continuing in subsequent decades. Its publication output peaked in the 1980s. Universität Freiburg displayed a remarkable research contribution, particularly in the 1980 s and 1990s. The institution maintained a consistent publication trend. Eberhard Karls Universität Tübingen showed a strong research output from the 1970 s through the 1990s. Ludwig-Maximilians-Universität München exhibited steady research productivity across multiple decades, with noticeable peaks in the 1980 s and 1990s. Universität Bonn maintained a consistent publication record, with a pronounced increase in research output in the late twentieth century and early twenty-first century. Universität Heidelberg displayed periodic peaks in research activity, with significant contributions spanning multiple decades. Justus-Liebig-Universität Gießen showed fluctuations in publication output but remained actively engaged in research over time. Hannover Medical School demonstrated a strong research trajectory in recent years. Thus, while some universities were very active in the past, new players are emerging.

At the international level, Germany was the dominant contributor, with 4852 publications, far surpassing all other countries. The USA ranked second, with 860 papers, followed by Japan (661 papers) and China (507 papers).

**Fig. 2:**
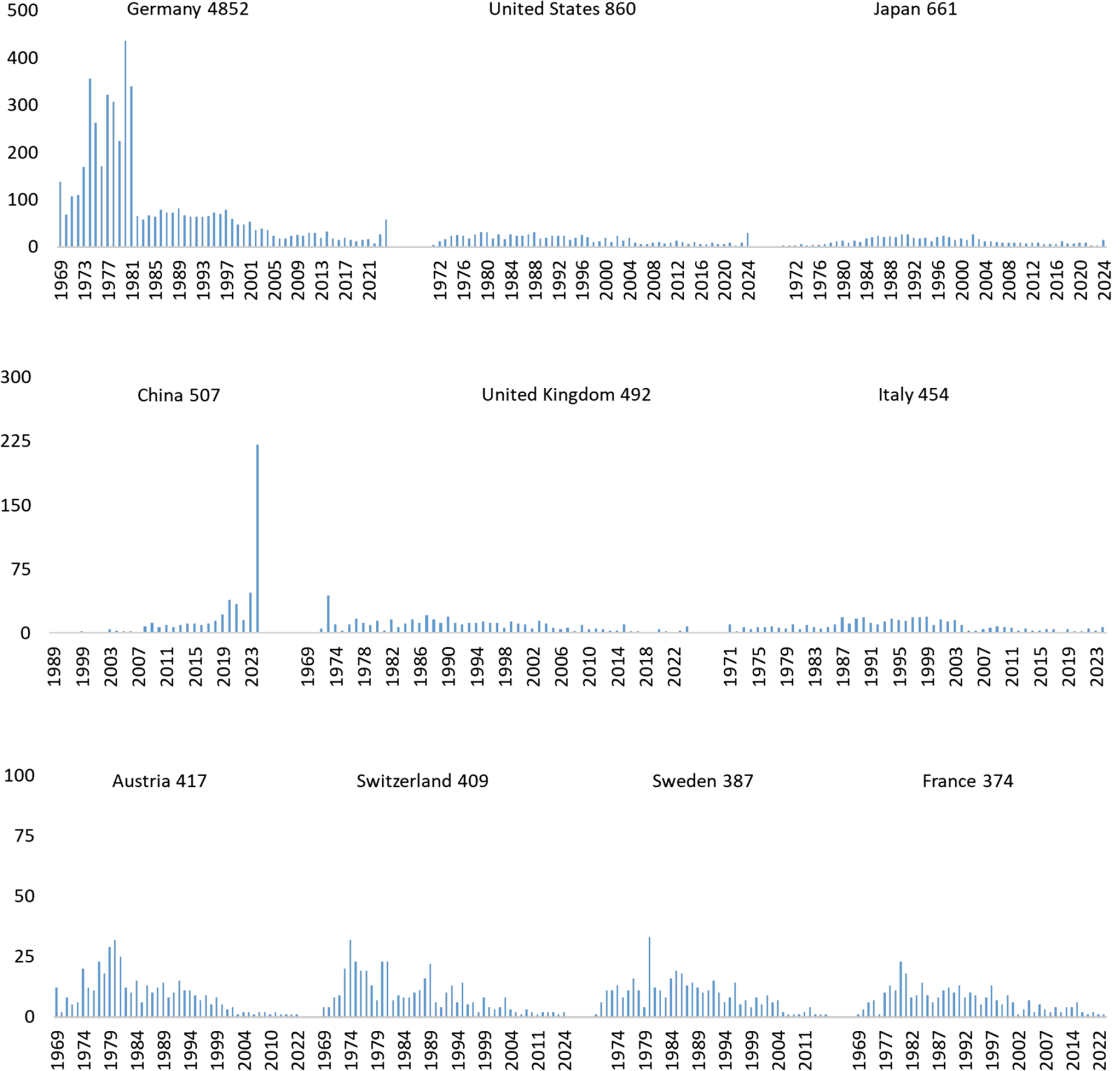
The Top Ten Countries’ Dynamics. Publication trends of the ten most active countries in *Naunyn–Schmiedeberg’s Archives of Pharmacology* (1969–2024), illustrating national research output and its evolution. The data is for all research articles

The publication dynamics of the top ten contributing countries in NSAP highlight distinct trends in research output over time (Fig. 2). Germany led the contributions with a total of 4852 publications, displaying strong productivity peaks in the 1970 s and 1980 s, particularly in 1974 (*n* = 356) and 1980 (*n* = 435), followed by a decline in the 1990 s and early 2000s. A recent resurgence occurred in 2024 (*n* = 58). The USA followed with 860 publications, with moderate yet steady contributions peaking in 1988 (*n* = 31) and 1980 (*n* = 31), before experiencing a decline in the 2000 s and a sharp rise in 2024 (*n* = 29). Japan contributed 661 research articles, showing steady growth through the 1980 s and 1990 s, with notable peaks in 1991 (*n* = 27) and 1990 (*n* = 26), while activity remained lower but consistent in recent years (2024, *n* = 15). China, with 507 publications, displayed a dramatically increasing trend, particularly post-2008. The highest contributions appeared in 2024 (*n* = 221), far exceeding previous peaks in 2023 (*n* = 47) and 2020 (*n* = 39). The UK (*n* = 492) had a strong early presence, peaking in 1973 (*n* = 44), but saw a decline in output after the early 2000 s, with minimal contributions in the 2010s. Italy (*n* = 454) maintained a steady output, particularly in the 1990 s, peaking in 1999 (*n* = 19) and 1998 (*n* = 18), followed by a decrease in recent years (2024, *n* = 7). Austria (*n* = 417) contributed significantly in the late twentieth century, especially in 1980 (*n* = 32), before experiencing a sharp decline post-2000. Switzerland (*n* = 409) also had a strong presence in the 1970 s and 1980 s, with a peak in 1974 (*n* = 32), but later output was sparse. Sweden (*n* = 387) showed consistent activity in the 1980 s, reaching a high in 1980 (*n* = 33), but contributions dropped sharply after 2000. The Netherlands (*n* = 323) followed a similar pattern, with a peak in 1980 (*n* = 23) and lower contributions post-2000. These publication trends reflect historical shifts in pharmacological research, national funding policies, and scientific priorities.

Regarding research funding, a threshold of at least 100 sponsored publications was established. The Deutsche Forschungsgemeinschaft (DFG) was the most prominent funding agency, supporting 341 papers, reinforcing Germany’s strong research foundation in pharmacology. The National Natural Science Foundation of China funded 167 papers, highlighting China’s growing influence in the field. Additionally, Brazilian funding agencies Conselho Nacional de Desenvolvimento Científico e Tecnológico (160 papers) and Coordenação de Aperfeiçoamento de Pessoal de Nível Superior (122 papers) played a crucial role in advancing research. The National Institutes of Health (NIH) in the USA supported 117 papers, further emphasizing its commitment to pharmacological studies. These findings underscored the significant contributions of individual researchers, institutions, countries, and funding sponsors in shaping the research landscape of NSAP over the years.

#### Descriptive data about the top 100 most cited research articles

The top 100 most cited research articles in NSAP were published between 1970 and 2006. The list of the top 100 most cited research articles is presented in supplementary Table 1. The citation data is presented in Table 3. These highly cited publications had an average age of 39.9 years, indicating their long-term impact on pharmacological research. The average number of citations per research article was 262.5, reflecting the significant influence of these papers in the field. The highest mean citations per research article were observed in 1984, where three research articles had an average of 456.67 citations, followed by 1979, with a mean of 392.20 citations across five research articles. Similarly, research articles published in 1986 had a high mean citation count of 384.00 per research article. These years represented peaks in the citation impact of research published in the journal.

**Table 3:** Annual citation metrics for the top 100 most cited research articles in *Naunyn–Schmiedeberg’s Archives of Pharmacology* (1969–2024), reflecting their citation impact over time

Year	Mean total citations per article	Number of publications	Mean total citations per year	Citable years
1970	218.00	1	3.89	56
1971	160.00	1	2.91	55
1972	362.33	3	6.71	54
1973	166.50	2	3.14	53
1974	220.00	3	4.23	52
1975	255.67	3	5.01	51
1976	290.57	7	5.81	50
1977	257.40	5	5.25	49
1978	229.67	3	4.78	48
1979	392.20	5	8.34	47
1980	210.00	3	4.57	46
1981	249.25	4	5.54	45
1982	264.00	6	6.00	44
1983	250.67	3	5.83	43
1984	456.67	3	10.87	42
1985	172.75	4	4.21	41
1986	384.00	3	9.60	40
1987	265.20	5	6.80	39
1988	216.57	7	5.70	38
1989	253.00	3	6.84	37
1990	226.50	2	6.29	36
1991	233.50	2	6.67	35
1993	215.00	2	6.52	33
1995	282.00	3	9.10	31
1996	166.00	1	5.53	30
1997	302.75	4	10.44	29
1999	203.50	2	7.54	27
2000	207.00	3	7.96	26
2001	235.00	1	9.40	25
2002	189.00	1	7.88	24
2003	230.00	1	10.00	23
2004	240.00	3	10.91	22
2006	304.00	1	15.20	20

In terms of citation rates per year, the most impactful research articles were from 2006, with a mean of 15.20 citations per year, followed by those from 2004 (10.91 per year) and 1984 (10.87 per year). Other high-impact years included 1997 (10.44 per year) and 2003 (10.00 per year), suggesting that research articles published in these periods continued to receive steady citations over time. The distribution of the most cited research articles showed that the 1970 s and 1980 s were particularly influential decades, with a consistent presence of highly cited papers. The 1990 s and early 2000 s also contributed significantly, with research articles maintaining high citation rates per year despite their relatively younger age compared to older publications.

Overall, the citation patterns of the top 100 most cited research articles demonstrated the long-term scholarly impact of research published in NSAP, with several decades contributing influential papers that remained relevant in pharmacological studies.

#### The top contributors (authors, universities, and countries) in the top 100 most cited research articles

A total of 314 authors contributed to the top 100 most cited research articles in NSAP. Only five authors published single-authored papers, indicating that nearly all publications were collaborative efforts. The average number of co-authors per document was 3.83, highlighting a strong trend toward teamwork in pharmacological research. Additionally, 15% of the research articles involved international co-authorship, demonstrating a notable level of global scientific collaboration.

The performance of authors was evaluated using multiple bibliometric indicators, including the number of publications (NP), total citations (TC), h-index, g-index, m-index, HG composite index, and Q index, along with the publication starting year (PY_start). Since a single metric might not fully represent an author’s impact, a combination of these indicators was used to provide a comprehensive assessment of author performance. The list of the top 50 authors (on the basis of total citations) is provided in supplementary Table 2. The data revealed notable differences across citation and productivity metrics. Schlicker E. led with 1870 total citations from six publications, achieving an h-index of 6, g-index of 6, m-index of 0.14, HG-composite of 6, and a Q2 Index of 0.92. Hoyer D. closely followed with 1,774 citations from six publications (h = 6, g = 6, m = 0.133, HG = 6, Q2 = 0.89). Despite publishing only three papers, Lembeck F. accumulated 1721 citations, maintaining an h-index and g-index of 3, an m-index of 0.064, HG-composite of 3, and Q2 Index of 0.44. Starke K. authored six papers garnering 1694 citations (h = 6, g = 6, m = 0.111, HG = 6, Q2 = 0.82), while Engel G. contributed five papers with 1542 citations (h = 5, g = 5, m = 0.111, HG = 5, Q2 = 0.74). The present data confirm that relying solely on a single metric does not provide a straightforward comparative message (Fox And Seifert 2024). Instead, employing multiple indicators—including h-index, g-index, m-index, HG-composite, and Q2 Index—offers a more comprehensive and balanced assessment of author performance (Hassan And Paas 2025).

Among the top 100 most cited papers in NSAP, only a few universities contributed at least five publications. Institut de Recherches Servier led the list with 10 papers, followed by Vernalis Research, which published six papers. Additionally, Toxikologische Abteilung der Universität Bonn, University of Bonn, and Universität Würzburg each contributed five publications, marking them as key institutions in highly cited pharmacological research.

The top 100 most cited papers in NSAP were authored by researchers from a diverse range of countries. Germany emerged as the most dominant contributor, with 32 publications. Sweden followed with 16 papers, while Switzerland contributed 12 publications, reinforcing the significance of European institutions in high-impact research. France and the USA each contributed 11 papers, demonstrating their substantial influence in the field. The UK produced seven highly cited papers, while Austria contributed six. Italy, with five publications, was also among the leading nations. Other countries with notable contributions included the Netherlands and Spain, each with four publications, while Canada followed with three papers. Countries with a smaller presence, each contributing a single paper, included Australia, Hungary, Ireland, Japan, and Portugal.

#### The main theme of the top 100 most cited research articles

To explore the main focus of the top 100 most cited papers, we analyzed the research topics.

## Enzyme inhibition and neurotransmitter synthesis

Research in the early 1970 s explored key enzymatic pathways involved in neurotransmission. Kukovetz and Poch (1970) highlighted cyclic-3′,5′-nucleotide-phosphodiesterase inhibition as a mechanism of action for vasodilators like papaverine. Complementarily, biochemical methodologies to assess neurotransmitter precursors were refined by Kehr et al. (1972) and Carlsson et al. (1972), who measured DOPA, tyrosine, and tryptophan hydroxylase activities. Atack and Lindqvist (1973) further developed fluorimetric assays for 5-hydroxyindoles, enhancing the quantification of serotonin-related compounds.

## Adrenergic, cholinergic, and neurotransmitter modulation

Several studies dissected the mechanisms of adrenergic and cholinergic signaling. Starke (1972) investigated α-sympathomimetic inhibition in cardiac tissue, while Starke et al. (1974) and Haeusler (1974) focused on clonidine’s modulation of sympathetic activity. Hedqvist and Fredholm (1976) revealed adenosine’s dual influence on adrenergic neurotransmission. Andén et al. (1976) categorized α-adrenoceptor subtypes responsible for differential pharmacologic responses.

## Dopaminergic neurotransmission and depression models

Dopaminergic regulation emerged as a central theme. Andén and Stock (1973) demonstrated the suppression of nigral dopamine neurons by GABA and GHB. Strömbom (1976) connected catecholaminergic activity to behavioral responses, while Walters and Roth (1976) introduced a model for analyzing presynaptic dopamine receptor interactions. Notably, Vetulani et al. (1976) proposed that reduced cyclic AMP sensitivity in the limbic forebrain underlies the action of various antidepressants.

## Drug mechanisms in cardiovascular and central nervous systems

Pharmacodynamic studies explored drug-receptor interactions in both the cardiovascular and nervous systems. Bayer et al. (1975) examined the electrophysiological actions of verapamil and D600 on heart cells. Capsaicin’s neurotoxic and desensitizing effects were established by Szolcsányi et al. (1975), and diazepam’s spinal site of action was characterized by Polc et al. (1974). Kohlhardt and Fleckenstein (1977) detailed nifedipine’s role as a calcium channel blocker in cardiac tissue.

## Neuropeptides and sensory signaling

Emerging interest in neuropeptides included the identification of substance P’s role in neurogenic vasodilation and plasma extravasation (Lembeck & Holzer 1979). Gamse et al. (1981) explored the selective depletion of neuropeptides like substance P and somatostatin following capsaicin administration, contributing to understanding nociception (Gamse 1982). Lee et al. (1982) proposed multiple receptor types for substance P, suggesting a complex signaling landscape.

## Receptor characterization and neuropharmacological advances

A substantial body of work was dedicated to identifying and differentiating receptor subtypes. Aghajanian and Bunney (1977) used single-cell recordings to study dopamine autoreceptors, while Starke et al. (1978) analyzed dopaminergic drug interactions in vitro. Carlsson and Lindqvist (1978) showed neurotransmitter synthesis depends on precursor availability. Studies by Timmermans et al. (1979) and Docherty and McGrath (1980) differentiated α-adrenoceptor subtypes based on pharmacological responses. Novel ligands such as [^125^Iodo]cyanopindolol enabled precise β-adrenoceptor mapping (Engel et al. 1981).

## Serotonin receptors and antidepressant pharmacology

Multiple studies dissected the role of serotonin in central nervous system (CNS) regulation and drug response. Martin and Sanders-Bush (1982) distinguished 5-HT_1_ and 5-HT_2_ binding sites, while Engel et al. (1983) linked presynaptic serotonin autoreceptors with inhibitory modulation. Fozard (1984) introduced MDL 72222 as a selective neuronal serotonin antagonist. Chaput et al. (1986) evaluated the effects of citalopram on 5-HT autoreceptor sensitivity. In contrast to traditional selective serotonin reuptake inhibitors (SSRIs), Mennini et al. (1987) found tianeptine to selectively enhance serotonin uptake, suggesting an alternative antidepressant mechanism.

## Monoamines and disease models

Schoemaker et al. (1985) linked [^3^H]cocaine binding at dopamine uptake sites to neurodegeneration in Parkinson’s disease models. O’Carroll et al. (1983) studied dopamine metabolism by monoamine oxidase, revealing regional specificity of its isoforms. Arbilla et al. (1985) characterized zolpidem’s effects at benzodiazepine receptors, and Markstein et al. (1986) identified adenylate cyclase stimulation via 5-HT_1A_ receptors in the hippocampus.

## Adenosine receptor characterization and signaling

Bruns et al. (1987) and Lohse et al. (1987) laid the foundation for characterizing adenosine A_1_ receptors using the selective antagonist 8-cyclopentyl-1,3-dipropylxanthine (DPCPX), establishing its high binding affinity and specificity. Further selectivity within the adenosine receptor family was explored with 2-chloro-N_6_-cyclopentyladenosine as an A_1_ agonist (Lohse et al. 1988). This receptor research extended into comparative pharmacology using Chinese hamster ovary cells (CHO cells) (Klotz et al. 1997) and antagonist studies of A_2A_ receptors (Ongini et al. 1999). Cunha et al. (1996) emphasized the regional specificity of A_2A_ receptors in the hippocampus and cortex, while Fang et al. (1999) and Hoffmann et al. (2004) contributed to understanding adenosine’s interaction with β-adrenergic systems and broader receptor pharmacology.

## Serotonin receptors and modulatory mechanisms

Serotonergic modulation received extensive attention, especially through characterization of 5-HT_1_, 5-HT_2_, 5-HT_4_, and 5-HT_7_ receptors. Hjorth and Magnusson (1988) showed 8-hydroxy-2-(di-n-propylamino)tetralin (8-OH-DPAT’s) selective action on 5-HT_1A_ autoreceptors, while Blier et al. (1988) and Le Poul et al. (1995) explored receptor adaptations following long-term antidepressant treatments. Dumuis et al. (1989) introduced 5-HT_4_ receptor agonists that stimulate adenylate cyclase. López-Giménez et al. (1997) and Knight et al. (2004) visualized and pharmacologically differentiated 5-HT_2A_, 5-HT_2B_, and 5-HT_2C_ receptors.

## Histaminergic and cannabinoid interactions in neurotransmission

The regulatory influence of histamine on neurotransmission was revealed by Schlicker et al. (1988, 1989), who demonstrated H_3_ receptor-mediated inhibition of serotonin and noradrenaline release in the cortex. Parallel developments in cannabinoid pharmacology showed that CB_1_ receptor activation inhibits long-term potentiation (Terranova et al. 1995) and serotonin release (Nakazi et al. 2000), suggesting potent cross-talk between endocannabinoid and monoaminergic systems. Kathmann et al. (2006) further showed that cannabidiol modulates μ- and δ-opioid receptors allosterically.

## Nitric oxide, inflammatory modulators, and vascular pharmacology

Busse et al. (1987) and Mülsch and Busse (1990) emphasized the endothelial role of nitric oxide (NO) in vascular tone regulation, later elaborated by Förstermann and Kleinert (1995 reviewed the isoforms of NO synthase and their control mechanisms. Posadas et al. (2000) showed co-regulation of cyclooxygenase-2 and inducible NO synthase during inflammation, linking these pathways in immune responses. Costa et al. (2004) demonstrated the oral anti-inflammatory effects of cannabidiol in rats, supporting its therapeutic potential.

## Methodological innovations in neurotransmitter measurement

Westerink et al. (1987, 1988) advanced in vivo microdialysis for measuring extracellular neurotransmitter levels in conscious rats, improving understanding of calcium-dependent dopamine and acetylcholine release. Santiago and Westerink (1990) compared microdialysis probe types for dopamine measurement, further refining this method.

## Receptor mapping and visualization

Significant work also focused on mapping receptor distribution. Bruinvels et al. (1993) compared 5-HT_1B_ and 5-HT_1D_ binding sites via autoradiography, while Dubocovich et al. (1997) distinguished melatonin receptor subtypes using selective ligands. Audinot et al. (2003) developed new selective MT_1_ and MT_2_ ligands to enhance research on melatonin signaling. Klapproth et al. (1997) revealed non-neuronal acetylcholine synthesis in epithelial cells, expanding cholinergic signaling beyond synaptic functions.

## Pharmacokinetics and drug transport mechanisms

Pharmacokinetic studies addressed how drugs are metabolized or transported. Kroemer et al. (1993) identified the cytochrome P_450_ enzymes metabolizing verapamil, and Fang et al. (1999) mapped risperidone metabolism via CYP2D6 and 3A4. Pauli-Magnus et al. (2001) showed how proton pump inhibitors interact with P-glycoprotein, implicating this transporter in bioavailability.

### Section summary

The top 100 most cited research articles focused on enzyme inhibition and neurotransmitter synthesis, especially concerning catecholamines and serotonin. Researchers extensively investigated adrenergic, dopaminergic, and serotonergic signaling pathways, along with receptor subtypes such as α-, β-, and 5-HT receptors. A significant emphasis was placed on drug mechanisms in the cardiovascular and central nervous systems, including calcium channel blockers and benzodiazepines. Additional themes included neuropeptide signaling, histamine and cannabinoid modulation, nitric oxide in vascular function, receptor mapping, and methodological innovations like microdialysis. Later studies explored pharmacokinetics and drug transport, enriching understanding of metabolism and bioavailability.

### Part two—analysis of reviews

#### The annual publication details

The number of published review articles exhibited substantial variation over the years. The first recorded review in this dataset appeared in 1973 with a single publication. Subsequent years saw sporadic contributions, with 3 reviews in 1994, 4 in 1995, and 2 in both 1996 and 1997. A gradual increase began in 1998 with 6 reviews, and by 2000, the number had risen to 11. These numbers support the notion that, in its core, NSAP has been a journal for original research.

Between 2001 and 2010, the annual number of reviews fluctuated, with notable peaks in 2005 (12 reviews), 2006 (20 reviews), and 2011 (18 reviews). The period from 2012 to 2020 showed a relatively steady trend, ranging from 6 to 16 reviews per year, except for 2021, which saw a notable increase to 21 reviews. The number of per year publications (for reviews) is presented in Table 4.

**Table 4:** Annual publication trends in *Naunyn–Schmiedeberg’s Archives of Pharmacology* (1969–2024), showing the number of reviews published per year

Year	Number of publications	Year	Number of publications
1973	1	2009	6
1994	3	2010	8
1995	4	2011	18
1996	2	2012	8
1997	2	2013	11
1998	6	2014	11
1999	3	2015	16
2000	11	2016	6
2001	1	2017	5
2002	5	2018	7
2003	1	2019	9
2004	10	2020	9
2005	12	2021	21
2006	20	2022	29
2007	13	2023	62
2008	13	2024	227

A significant rise in review publications occurred in the most recent years. In 2022, the number of reviews reached 29, followed by a substantial increase to 62 reviews in 2023. The most remarkable growth was observed in 2024, with 227 reviews published, marking an unprecedented surge in review research articles in the journal’s history.

#### The top contributors

In this part the details about the top contributors (authors, universities, countries, and sponsors) are provided in Table 5. The most prolific author in publishing reviews within the journal was Michel, M.C., who contributed 26 reviews. Other notable contributors included Sharifi-Rad, J. (13 reviews), Tuli, H.S. (13 reviews), Batiha, G.E.S. (12 reviews), and Seifert, R. (12 reviews). Additionally, Calina, D. and Hosseinzadeh, H. each authored 11 reviews, while Al-Kuraishy, H.M. published 10 reviews.

**Table 5:** The most prolific contributors to *Naunyn–Schmiedeberg’s Archives of Pharmacology* (1969–2024) reviews, ranked by the total number of published articles

S#	Author name	Number of publications (at least 10)
1	Michel, M.C	26
2	Sharifi-Rad, J	13
3	Tuli, H.S	13
4	Batiha, G.E.S	12
5	Seifert, R	12
6	Calina, D	11
7	Hosseinzadeh, H	11
8	Al-kuraishy, H.M	10
S#	Affiliation	Number of publications (at least 10)
1	Mashhad University of Medical Sciences	31
2	Al-Azhar University	17
3	Maharishi Markandeshwar Deemed to be University, Mullana	17
4	Hannover Medical School	15
5	Chandigarh University	15
6	Johannes Gutenberg-Universität Mainz	14
7	Damanhour University	13
8	Institute of Nano Science and Technology, Mohali	13
9	Tehran University of Medical Sciences	12
10	Amsterdam UMC—University of Amsterdam	11
S#	Country	Number of publications (at least 30)
1	India	142
2	Germany	140
3	USA	67
4	Iran	63
5	China	50
6	Egypt	45
7	Saudi Arabia	38
8	Australia	31
S#	Funding sponsor	Number of publications (at least 10)
1	UK Research and Innovation	47
2	Deutsche Forschungsgemeinschaft	34
3	National Natural Science Foundation of China	20
4	Ministry of Science and Technology of the People's Republic of China	14
5	National Institutes of Health	14
6	Mashhad University of Medical Sciences	12
7	Bundesministerium für Bildung und Forschung	10

The publication trends of the most prolific authors demonstrated varied temporal contributions (supplementary Fig. 3). Michel, M.C. had a consistent presence, publishing reviews as early as 1995 and maintaining contributions across multiple years, with peaks in 2006 (2 reviews), 2008 (3 reviews), and 2022 (3 reviews). Seifert, R. had scattered contributions, notably in 2002, 2004, 2013, 2014, and 2015, 2023, and 2024.

A significant surge in review publications was observed in 2023 and 2024, particularly among newer prolific authors. Sharifi-Rad, J. (13 reviews in 2024), Tuli, H.S. (9 reviews in 2023, 4 in 2024), Batiha, G.E.S. (6 reviews in 2023, 4 in 2024), and Al-Kuraishy, H.M. (4 reviews in 2023, 4 in 2024) all exhibited a sharp rise in recent years. Similarly, Calina, D., who had no prior contributions before 2024, published 11 reviews in that year. Hosseinzadeh, H. maintained steady but increasing contributions, with notable outputs in 2016, 2020, 2023, and 2024.

Several institutions played a key role in contributing review publications to the journal. The Mashhad University of Medical Sciences led with 31 reviews, followed by Al-Azhar University and Maharishi Markandeshwar Deemed to be University, Mullana, each with 17 reviews. Other major contributors included Hannover Medical School (15 reviews), Chandigarh University (15 reviews), and Johannes Gutenberg-Universität Mainz (14 reviews). Additionally, Damanhour University and the Institute of Nano Science and Technology, Mohali, both contributed 13 reviews, while Tehran University of Medical Sciences (12 reviews) and Amsterdam UMC—University of Amsterdam (11 reviews) also had notable contributions.

The contributions of various institutions to review publications in Naunyn–Schmiedeberg’s Archives of Pharmacology evolved over time (supplementary Fig. 4). Among the leading institutions, Mashhad University of Medical Sciences demonstrated significant growth, contributing no publications before 2016 but rising to 18 in 2024. Similarly, Al-Azhar University had no recorded contributions until 2023, when it published one review, followed by a substantial increase to 16 in 2024. Maharishi Markandeshwar Deemed to be University, Mullana entered in 2023 with 11 reviews and maintained output in 2024 with 6 publications. Hannover Medical School exhibited sporadic contributions since 2009, peaking at 3 reviews in 2015 and contributing one review in 2024. Chandigarh University only appeared in 2023 with 5 reviews, increasing to 10 in 2024.

Other major contributors included Johannes Gutenberg-Universität Mainz, which first appeared in 2013 and produced 4 reviews in 2024, and Damanhour University, with minimal activity until 2023, when it published 6 reviews, followed by 4 in 2024. Institute of Nano Science and Technology, Mohali emerged in 2023 with 5 reviews, increasing to 8 in 2024. Tehran University of Medical Sciences had sporadic contributions, peaking at 3 in 2021, 2022, and 2023, and maintaining the same level in 2024. Amsterdam UMC—University of Amsterdam had minor contributions between 2006 and 2011 but did not record any publications after 2014.

A country-wise analysis showed that India led with the highest number of review publications (142 reviews), followed closely by Germany (140 reviews). The USA (67 reviews) and Iran (63 reviews) also had significant contributions. Other leading countries included China (50 reviews), Egypt (45 reviews), Saudi Arabia (38 reviews), and Australia (31 reviews).

Several funding agencies played a crucial role in supporting the publication of reviews in the journal. UK Research and Innovation was the most prominent funding sponsor, supporting 47 review research articles. The Deutsche Forschungsgemeinschaft contributed to 34 reviews, while the National Natural Science Foundation of China supported 20. Additionally, the Ministry of Science and Technology of the People’s Republic of China and the National Institutes of Health each funded 14 reviews. Other notable funding agencies included Mashhad University of Medical Sciences (12 reviews) and Bundesministerium für Bildung und Forschung (10 reviews).

**Fig. 3:**
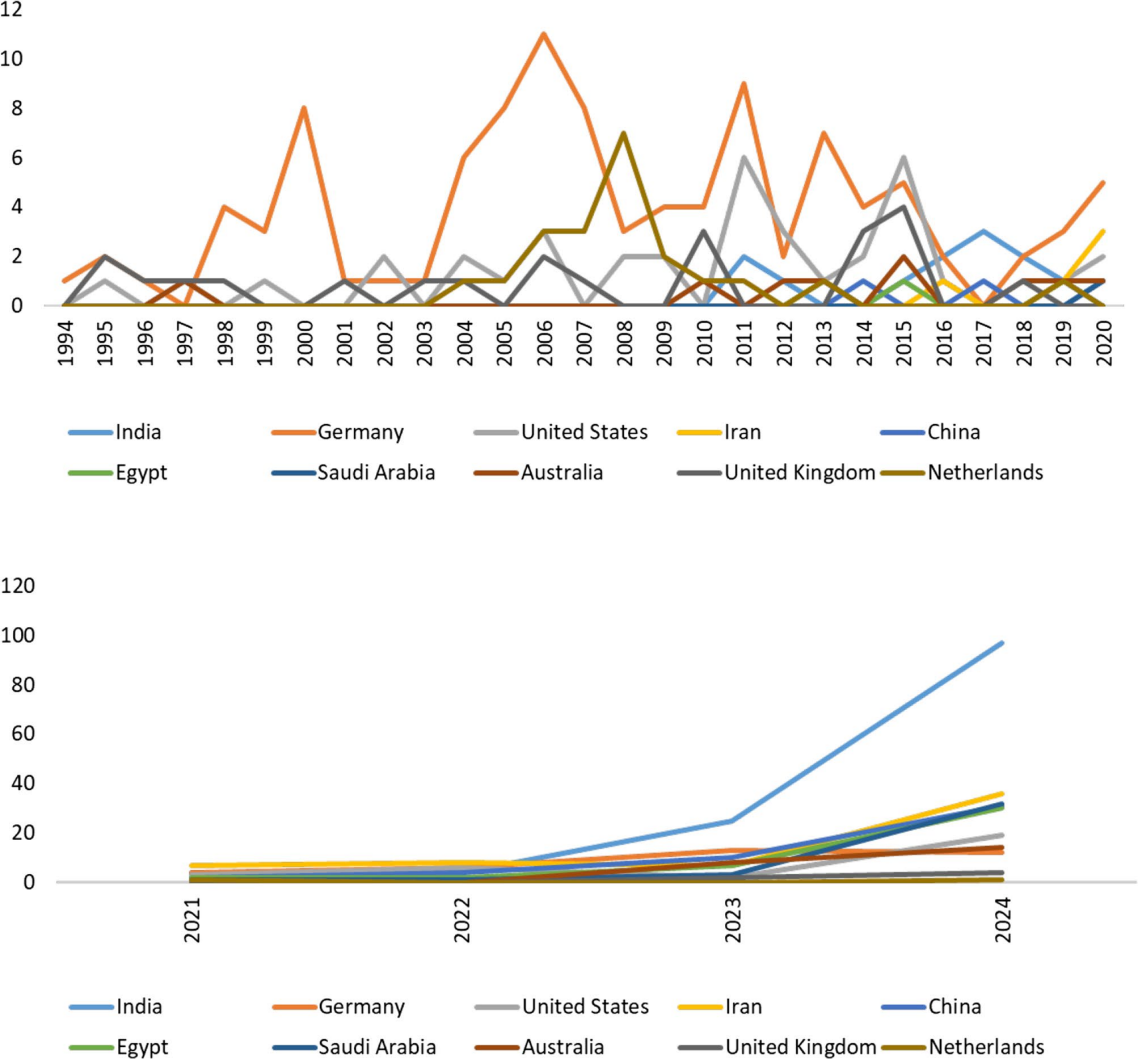
The Top Ten Countries’ Dynamics. Publication trends of the ten most active countries in *Naunyn–Schmiedeberg’s Archives of Pharmacology* (1969–2024), illustrating national research output and its evolution. The data is for all reviews

Country-wise contributions (in reviews) showed significant variations over the years (Fig. 3). Germany maintained a consistent presence since 1994, frequently ranking among the top contributors, with a peak of 13 publications in 2023. India demonstrated a dramatic increase in publications, contributing just a few papers per year before 2021 but surging to 25 in 2023 and reaching a leading position with 97 publications in 2024. The USA had moderate and fluctuating output, with a maximum of 19 publications in 2024. Iran exhibited steady growth, increasing from 7 reviews in 2021 to 36 in 2024. China followed a similar trend, rising from 1 to 2 annual publications before 2021 to 31 in 2024.

Among other countries, Egypt remained a moderate contributor, increasing its presence to 30 publications in 2024. Saudi Arabia showed modest growth, reaching 32 in 2024. Australia, with minimal contributions before 2023, reached 14 publications in 2024. The UK and the Netherlands had lower contributions compared to other nations, with 4 and 1 publication in 2024, respectively.

#### Descriptive data about the top 100 most cited reviews

The analysis of the top 100 most cited reviews from *Naunyn–Schmiedeberg’s Archives of Pharmacology* over the period 1973 to 2023 revealed key insights regarding publication trends, citation patterns, and document age. The list of the top 100 most cited reviews is provided in supplementary Table 3. The overall annual growth rate of these publications was 1.4%, indicating a steady increase in the number of reviews published. The average age of the documents was 17.9 years, suggesting that older works continue to contribute significantly to citations. On average, each document received 132.2 citations, highlighting the substantial influence of these reviews within pharmacology.

Review citation dynamics varied significantly across the years. In the early years (1973 to 1990 s), citation counts were generally low, with notable spikes in some years. The annual citation data for the top 100 most cited reviews is depicted in Table 6. For instance, in 1973, a single review document had an average of 57 citations. By 1994 and 1995, the mean citations per research article rose to around 127, with a total of three research articles each year contributing to these figures. Citation trends continued to grow in the subsequent years, especially around 2000, where the average citations per research article reached a remarkable 277.82 in a year that saw 11 reviews published. The year 2002 marked an extraordinary peak, with one review receiving 545 citations, a clear outlier for the dataset.

**Table 6:** Annual citation metrics for the top 100 most cited reviews in *Naunyn–Schmiedeberg’s Archives of Pharmacology* (1969–2024), reflecting their citation impact over time

Year	Number of publications	Mean total citations per article	Mean total citations per year	Citable years
1973	1	57.00	1.08	53
1994	3	127.00	3.97	32
1995	3	127.33	4.11	31
1996	2	52.00	1.73	30
1997	2	161.00	5.55	29
1998	4	144.00	5.14	28
1999	1	63.00	2.33	27
2000	11	277.82	10.69	26
2001	1	112.00	4.48	25
2002	1	545.00	22.71	24
2004	6	246.00	11.18	22
2005	5	85.40	4.07	21
2006	9	122.22	6.11	20
2007	9	116.33	6.12	19
2008	6	77.50	4.31	18
2009	1	267.00	15.71	17
2010	3	88.33	5.52	16
2011	2	82.50	5.50	15
2012	4	61.25	4.38	14
2013	2	78.50	6.04	13
2014	3	152.67	12.72	12
2015	4	80.50	7.32	11
2016	3	107.00	10.70	10
2017	1	41.00	4.56	9
2019	3	97.00	13.86	7
2020	3	75.33	12.55	6
2021	3	53.67	10.73	5
2022	2	45.00	11.25	4
2023	2	50.00	16.67	3

From 2005 onwards, citation rates began to show variability. For example, 2005 had five research articles with an average of 85.4 citations, while 2006 and 2007 saw relatively high citation counts of around 116.33 and 122.22, respectively, driven by nine reviews each year. Citation levels began to stabilize in the later years, with 2010 averaging 88.33 citations per research article. Notably, a sharp decline was observed by 2017, with one research article in that year receiving only 41 citations, reflecting a general decrease in citation rates for the reviews published after the peak years of the early 2000s. Over the more recent years, including 2020 to 2023, citation counts remained relatively moderate. In 2023, the mean citation per research article was 50, suggesting a slight resurgence in interest. In terms of citable years, the reviews generally enjoyed long lifespans. For example, the review published in 1973 remained citable for 53 years, which is consistent with the long-lasting relevance of high-impact reviews.

#### The top authors in the top 100 most cited reviews

Among the top 100 most cited reviews in Naunyn–Schmiedeberg’s Archives of Pharmacology, a total of 268 authors contributed to these influential works. Of these, 23 authors published single-authored reviews, reflecting a balance between individual and collaborative efforts in pharmacology research. The level of collaboration among authors was notable, with an average of three co-authors per document. International co-authorships accounted for 22% of the publications, demonstrating a moderate level of cross-border scientific collaboration.

We also explored the top contributing authors, recognizing that a single indicator is insufficient to comprehensively evaluate an author’s impact. To provide a more detailed analysis of author performance, we used multiple bibliometric indicators, including total citations, number of publications, *h*-index, *g*-index, *m*-index, Hg composite index, Q2 index, and the starting year of an author’s publication activity. These metrics collectively offered insights into productivity, citation influence, and long-term scholarly contributions within the journal. The list of top 50 most productive authors in reviews is provided in supplementary Table 4. The analysis highlighted distinct patterns in citation impact and productivity indicators. Michel MC stood out as the leading author, accumulating 898 total citations from seven publications since 1995, with an h-index and g-index of 7, m-index of 0.226, HG-composite score of 7, and a Q2 Index of 1.26. In contrast, Zimmermann H**,** despite publishing only one highly cited review since 2000, garnered 862 citations (h = 1, g = 1, m = 0.038, HG = 1, Q2 = 0.19). Eichelbaum M also authored a single review article starting in 2004, which received 751 citations (h = 1, g = 1, m = 0.045, HG = 1, Q2 = 0.21).

These findings confirm that reliance on a single indicator, such as total citations or the h-index alone, does not convey a clear comparative message. The use of a range of indicators provides a more comprehensive assessment of author performance and research influence.

#### The main themes of the top 100 most cited reviews

The main thematic focus of several key reviews is summarized below. The top-cited reviews cover numerous diverse areas of pharmacology, covering more fields than the top-cited research papers. This shows that decisions of authors to submit a research paper or a review paper to NSAP are independent of each other and that NSAP is valued much as a venue for reviews. Many authors contributed reviews to NSAP who with respect to research papers were less prolific. Traditionally, NSAP has been used as a platform for emerging senior scientists to leave their mark as authorities in their research fields. The flexible approach of NSAP to review papers in terms of structure, length, and topic (Research First-policy) has rendered the journal very attractive for reviews because different research fields need different approaches and formats. NSAP will continue this tradition of flexible-format review papers in the future.

One of the journal’s most influential reviews is by Zimmermann (2000), titled “Extracellular metabolism of ATP and other nucleotides.” This seminal research article provided a comprehensive overview of the enzymatic breakdown of extracellular nucleotides, a crucial component of purinergic signaling. Zimmermann’s detailed analysis of ectonucleotidases laid the groundwork for subsequent research on ATP-mediated signaling pathways in both physiological and pathological conditions. Zanger et al. (2004) published a highly regarded review on cytochrome P_450_ 2D6. Their work offered an in-depth exploration of CYP2D6 polymorphisms, their impact on drug metabolism, and clinical implications. This review has become a foundational resource in pharmacogenomics, helping clinicians and researchers appreciate the complexities of interindividual drug response. Seifert and Wenzel-Seifert (2002) examined the constitutive activity of G-protein-coupled receptors (GPCRs) and its role in physiology and disease. The authors articulated how constitutively active GPCRs can act as disease drivers, emphasizing the need for inverse agonists as therapeutic agents. Fredholm et al. (2000) offered a detailed characterization of adenosine receptors and their genes. This review mapped receptor subtypes (A_1_, A_2A_, A_2B_, A_3_), their tissue distribution, and functional relevance, serving as a cornerstone for researchers exploring adenosine-mediated signaling and its pharmacological targeting.

Von Kügelgen and Wetter (2000) focused on P_2Y_ receptors, a subset of purinergic GPCRs. Their molecular pharmacology analysis of P_2Y_ subtypes significantly contributed to drug development targeting thrombosis, inflammation, and immune regulation. Piechota-Polanczyk and Fichna (2014) explored the role of oxidative stress in inflammatory bowel diseases. The authors synthesized evidence linking oxidative damage to gastrointestinal inflammation, emphasizing antioxidant-based therapies—a theme resonating with current trends in inflammation research. Chatterjee (2007) provided a landmark synthesis on the pharmacological strategies targeting renal ischemia–reperfusion injury (IRI), a condition central to acute kidney injury. The review offered mechanistic insights into oxidative stress, inflammation, and endothelial dysfunction, and proposed therapeutic potential for agents such as statins, nitric oxide donors, and antioxidants. Its translational importance is well recognized. Michel et al. (1995) delivered a foundational review categorizing α_1_-adrenoceptor subtypes, a critical pharmacological topic for cardiovascular and urogenital therapy. The review bridged pharmacodynamics with receptor biology, influencing drug classification and receptor-targeted therapies for years.

Gawel et al. (2019) addressed the need for standardization in behavioral pharmacology by analyzing the Barnes maze task. The review detailed experimental variables and data interpretation issues in rodent models of spatial learning, offering essential guidance for neuropharmacological studies. Kaumann and Molenaar (1997) explored the complex role of β-adrenoceptor subtypes in human cardiac function. Their integrative review influenced both basic cardiology research and clinical drug design targeting heart failure and arrhythmias. Villalón and Centurión (2007) reviewed the diverse cardiovascular actions of serotonin, emphasizing the involvement of multiple 5-HT receptor subtypes. This pharmacological update informed therapeutic strategies in vascular diseases and migraine.

Lambrecht (2000) presented one of the earliest comprehensive profiles on agonists and antagonists of P_2X_ receptors, highlighting their role in pain and inflammation. This review remains a reference point for drug development targeting purinergic signaling. Avelino and Cruz (2006) examined the expression and function of TRPV1 in the urinary tract, discussing its implications in disorders like overactive bladder and interstitial cystitis. The clinical relevance of this channel contributed to the paper’s sustained influence. Geyer et al. (2006) highlighted the broader roles of the SLC10 family beyond bile acid transport, with implications for drug absorption and metabolic diseases. This evolutionary and functional overview gained considerable attention. Lovinger (1997) offered a conceptual framework on how alcohol modulates neurotransmitter-gated ion channels, focusing on GABA and NMDA receptors. This influential review guided subsequent research on the neurobiology of addiction. Bedi et al. (2016) explored the pleiotropic effects of HMG-CoA reductase inhibitors (“statins”), such as anti-inflammatory and antioxidant properties, proposing new therapeutic avenues in neurodegeneration, cancer, and renal disease. The wide-reaching potential of statins is reflected in this work. Dietrich et al. (2005) presented a detailed review on the TRPC3/6/7 subfamily of cation channels, emphasizing both their functional characterization and physiological relevance. Gross et al. (2013) examined the therapeutic relevance of dopamine D_3_ receptor antagonism in schizophrenia, indicating that despite advancements in pharmacotherapy, D_3_ antagonists may still warrant consideration. Petzinger and Geyer (2006) reviewed drug transporters with respect to pharmacokinetics, focusing on how these transport systems influence drug absorption, distribution, metabolism, and excretion. Mayer and Andrew (1998) focused on nitric oxide synthases, specifically describing their catalytic function and summarizing progress in developing selective inhibitors. Gavioli and Calò (2006) reviewed ligands of the nociceptin/orphanin FQ receptor, highlighting their antidepressant- and anxiolytic-like effects. In the cardiovascular domain, Savelieva et al. (2010) evaluated evidence for the primary and secondary prevention of atrial fibrillation using statins and polyunsaturated fatty acids. Complementing this, Dhein et al. (2010) discussed how improving cardiac gap junction communication through antiarrhythmic peptides could represent a novel antiarrhythmic mechanism. Offermanns and Schultz (1994) analyzed how G protein-based transmembrane signaling systems process complex information. In urology-focused pharmacology, Igawa and Michel (2013) presented the pharmacological profiles of β_3_-adrenoceptor agonists under clinical development for treating overactive bladder syndrome. Campian et al. (2006) evaluated the validity of animal models used to study treatments for pulmonary hypertension.

Ghasemzadeh Rahbardar and Hosseinzadeh (2020) compiled updated findings on the effects of rosmarinic acid in nervous system disorders. McNulty et al. (2015) discussed the potential of targeting TRPV4 as a therapeutic strategy for joint diseases. In the context of nanotechnology, Andra et al. (2019) reviewed the pharmaceutical applications of phytosynthesized metal oxide nano research articles, while their later work (2021) focused on emerging nanomaterials for antibacterial textile fabrication. Vauquelin et al. (2012) analyzed atypical antipsychotics, particularly clozapine, and addressed the therapeutic benefits of fast-off D_2_ dopamine receptor antagonism. Anand et al. (2012) explored the expanding pathophysiological roles of mast cells beyond allergic responses. Plant and Schaefer (2005) investigated receptor-operated cation channels formed by TRPC4 and TRPC5. Humphrey et al. (1995) provided insights into P2X purinoceptors, and Bonvini et al. (2015) addressed the therapeutic targeting of TRP channels for chronic cough. Finally, Gudermann et al. (2000) reviewed the contribution of receptor/G protein signaling to cell growth and transformation.

## Limitations

Although this study presented the top contributors—especially authors—to Naunyn–Schmiedeberg’s Archives of Pharmacology, no manual verification was performed to confirm the accuracy of author names. Issues such as name variations, use of initials, or spelling errors were not corrected, which may affect the accuracy of author productivity data. We analyzed the focus of the top 100 most cited research articles and reviews using title-based co-word analysis. However, the full texts of these documents were not reviewed. As a result, some important findings or contributions may have been missed. This is a bibliometric study and not a qualitative analysis. While it captures patterns in publication trends, citation impact, and collaboration, it does not explore how the most cited papers contributed in terms of research content or clinical relevance.

## Editorial implications

The increasing internationalization of authorship in NSAP has called for recent changes in the composition of the editorial board. While traditionally, the journal has always had a focus on German pharmacologists serving as editors, recently, leading pharmacologists from India, China, Iran, and Egypt have been appointed. This process is not yet completed and will continue. The new appointments, on one hand, reflect recognition of the quality of the contributions of these countries to NSAP. On the other hand, these appointments have the function of ensuring high scientific quality of submissions from these countries so that the published papers are read and cited appropriately. In addition, the scientific input of these editors will shape the future scope of the topics in the journal.

This study has shown that a high-profile but narrow scope of a journal for research papers can lead to long-lasting problems if, within a short time frame, leading contributors retire. These thematic problems cannot be resolved within a short period of time. Thus, thematic diversity will ensure the viability of the journal when new topics emerge. Therefore, recently, NSAP has implemented a number of new “Collections and calls for papers” on diverse and current scientific topics that are different from traditional subsections of pharmacology journals (https://link.springer.com/journal/210/collections?filter=Open). Readers and authors are cordially invited to make contributions for additional “Collections and calls for papers.” We want to ensure that we capture emerging topics in pharmacology rapidly and give these topics high visibility.

Traditionally, the number of reviews published in NSAP was rather low. Since in general, reviews tend to attract many citations, the recent strong increase in review submissions and review publications was initially welcome. However, once we have become aware of serious research integrity issues with some of the recently published reviews (Seifert et al. 2025), we have immediately implemented very strict editorial guidelines (effective March 2025) to prevent the future publication of questionable review papers. Possibly, non-declared use of large language models (LLM), also referred to as “artificial intelligence” played a role in the recent review integrity cases. The respective reviews have been swiftly retracted. For NSAP, this represents an unprecedented situation since so far, not a single review in the journal has been retracted. Traditionally, reviews are written by internationally recognized experts in the field with no doubt about their integrity.

Any author who wishes to submit a review to NSAP must follow the following rules (https://link.springer.com/journal/210/submission-guidelines#Instructions%20for%20Authors_Important%20Submission%20Policy):

1. In the cover letter, the authors must state why the topic chosen is of relevance for the readership of NSAP.
2. In the cover letter, the authors must describe the originality and timeliness of the review.
3. In the cover letter, the authors must document their expertise and track record in the field.
4. In the cover letter, the authors must list (with full bibliography including authors, title, journal, volume, pages, year and digital object identifier (doi)) at minimum 3 representative publications related to the content of the review. Incomplete bibliography or global links to google scholar profiles are not accepted.

Review submissions not abiding to these rules will be rejected without review. No exceptions will be made.

In addition, should a review become accepted for publication, the authors must state under “Declarations”:

## The authors confirm that no paper mill and no artificial intelligence was used

These editorial guidelines are strictly implemented and serve several complementary functions:

1. Should the Editor-in-Chief become aware of the use of artificial intelligence in an already published review, swift retraction will follow.
2. Reviews have a huge impact on a scientific field and are often used by newcomers as orientation. Therefore, reviews written by non-experts can lead to a tremendous waste of human, laboratory, and time resources, even jeopardizing academic careers, particularly of young and unexperienced scientists. These risks must be avoided at all cost, and NSAP will make its contribution to risk avoidance.
3. NSAP must and will protect the integrity of the large number of high-impact reviews published in the journal, none of which before 2024 has ever been retracted so far.
4. NSAP must and will protect the integrity of those review authors who follow the editorial guidelines and make important and honest academic contributions to a scientific field. LLMs lack the ability for critical data analysis and development of novel scientific concepts and undermine the proper assessment of the scientific authority of researchers writing reviews.
5. For the assessment of tenure and promotion, review articles play an important role, demonstrating the establishment of a scientist as “authority” in a given field. This important academic assessment tool must not be annihilated by individuals inappropriately trying to establish “(pseudo)authority” by producing LLM-generated reviews.
6. NSAP will make its contribution to prevent the contamination of the scientific literature with questionable reviews that then feed again into the “learning material” of LLMs and further distort the presentation of a scientific field.
7. In a broader societal context, preventing the publication of LLM-generated reviews is important for maintaining the credibility of science. The use of LLM in reviews by scientists could be used by anti-science movements as an argument that these individuals are not productive anyway and lack authentic ideas. Thus, the use of LLM in reviews could ultimately result in cuts to science funding.

These strict editorial guidelines may appear inadequate, harsh, unrealistic, or even “old-fashioned” to some individuals, but from the now 3-month experience with these guidelines, we can conclude that they seem to be effective at selecting honest academic review papers.

## Conclusions

Our bibliographic analysis of research papers revealed that NSAP, over the past 50 years, was very strong in some selected fields of pharmacology. However, this focus made the journal vulnerable to changes in research topics and loss of high-profile authors because of retirement. Consequently, NSAP has diversified its thematic portfolio as highlighted by the recent implementation of “Collections and calls for papers.”

Our bibliographic analysis also revealed an unprecedented surge of reviews in recent years, passing peer review uneventfully. This wave of reviews coincides with the advent of powerful LLMs. While our analysis was not designed to unmask the potential undeclared use of LLM in recent reviews, we have taken numerous editorial measures to ensure that in the future, reviews published in NSAP remain a trustworthy source of information and guide for future research.

## Supplementary Information

Below is the link to the electronic supplementary material.

Supplementary file 1 (DOCX 106 KB)

Supplementary file 2 (DOCX 696 KB)

## Data Availability

The source data for this study are available upon reasonable request.
