# Supplementary material for: A bibliometric study of the most-cited research articles and reviews in Naunyn–Schmiedeberg’s Archives of Pharmacology (1969–2024)
Source: Naunyn Schmiedebergs Arch Pharmacol. 2025 Aug 1;399(1):1211–33. doi: 10.1007/s00210-025-04471-7 (PMC12894139; doi:10.1007/s00210-025-04471-7)
Supplement: Supplementary file 1 — (DOCX 106 KB) [file 210_2025_4471_MOESM1_ESM.docx]

**Legends**

**Supplementary Table 1:** The list of **top 100 most cited research articles** in Naunyn-Schmiedeberg’s Archives of Pharmacology (1969–2024), ranked by total citation count.

**Supplementary Table 2:** The 50 most productive authors in the **top 100 most cited research articles** of *Naunyn-Schmiedeberg’s Archives of Pharmacology* (1969–2024), ranked based on total citations.

**Supplementary Table 3:** The list of **top 100 most cited reviews** in Naunyn-Schmiedeberg’s Archives of Pharmacology (1969–2024), ranked by total citation count.

**Supplementary Table 4:** The 50 most productive authors in the **top 100 most cited reviews** of *Naunyn-Schmiedeberg’s Archives of Pharmacology* (1969–2024), ranked based on total citations.

| **S#** | **Authors** | **Title** | **Year** | **Volume** | **Issue** | **Page Start** | **Page End** | **Cited By** |
| --- | --- | --- | --- | --- | --- | --- | --- | --- |
| **1** | Lembeck F.; Holzer P. | Substance P as neurogenic mediator of antidromic vasodilation and neurogenic plasma extravasation | 1979 | 310 | 2 | 175 | 183 | 1057 |
| **2** | Handley S.L.; Mithani S. | Effects of alpha-adrenoceptor agonists and antagonists in a maze-exploration model of 'fear'-motivated behaviour | 1984 | 327 | 1 | 1 | 5 | 820 |
| **3** | Engel G.; Göthert M.; Hoyer D.; Schlicker E.; Hillenbrand K. | Identity of inhibitory presynaptic 5-hydroxytryptamine (5-HT) autoreceptors in the rat brain cortex with 5-HT1B binding sites | 1986 | 332 | 1 | 1 | 7 | 640 |
| **4** | Carlsson A.; Davis J.N.; Kehr W.; Lindqvist M.; Atack C.V. | Simultaneous measurement of tyrosine and tryptophan hydroxylase activities in brain in Vivo using an inhibitor of the aromatic amino acid decarboxylase | 1972 | 275 | 2 | 153 | 168 | 604 |
| **5** | Klotz K.-N.; Hessling J.; Hegler J.; Owman C.; Kull B.; Fredholm B.B.; Lohse M.J. | Comparative pharmacology of human adenosine receptor subtypes - Characterization of stably transfected receptors in CHO cells | 1997 | 357 | 1 | 1 | 9 | 486 |
| **6** | Magnan J.; Paterson S.J.; Tavani A.; Kosterlitz H.W. | The binding spectrum of narcotic analgesic drugs with different agonist and antagonist properties | 1982 | 319 | 3 | 197 | 205 | 450 |
| **7** | Aghajanian G.K.; Bunney B.S. | Dopamine "Autoreceptors": Pharmacological characterization by microiontophoretic single cell recording studies | 1977 | 297 | 1 | 1 | 7 | 427 |
| **8** | Förstermann U.; Kleinert H. | Nitric oxide synthase: expression and expressional control of the three isoforms | 1995 | 352 | 4 | 351 | 364 | 426 |
| **9** | Starke K.; Endo T.; Taube H.D. | Relative pre- and postsynaptic potencies of α-adrenoceptor agonists in the rabbit pulmonary artery | 1975 | 291 | 1 | 55 | 78 | 415 |
| **10** | Fozard J.R. | MDL 72222: a potent and highly selective antagonist at neuronal 5-hydroxytryptamine receptors | 1984 | 326 | 1 | 36 | 44 | 389 |
| **11** | Lohse M.J.; Klotz K.-N.; Lindenborn-Fotinos J.; Reddington M.; Schwabe U.; Olsson R.A. | 8-Cyclopentyl-1,3-dipropylxanthine (DPCPX) - a selective high affinity antagonist radioligand for A1 adenosine receptors | 1987 | 336 | 2 | 204 | 210 | 389 |
| **12** | Saria A.; Lundberg J.M.; Skofitsch G.; Lembeck F. | Vascular protein leakage in various tissues induced by substance P, capsaicin, bradykinin, serotonin, histamine and by antigen challenge | 1983 | 324 | 3 | 212 | 218 | 368 |
| **13** | Andén N.-E.; Grabowska M.; Strömbom U. | Different alpha-adrenoreceptors in the central nervous system mediating biochemical and functional effects of clonidine and receptor blocking agents | 1976 | 292 | 1 | 43 | 52 | 358 |
| **14** | Bruns R.F.; Fergus J.H.; Badger E.W.; Bristol J.A.; Santay L.A.; Hartman J.D.; Hays S.J.; Huang C.C. | Binding of the A1-selective adenosine antagonist 8-cyclopentyl-1,3-dipropylxanthine to rat brain membranes | 1987 | 335 | 1 | 59 | 63 | 357 |
| **15** | Vetulani J.; Stawarz R.J.; Dingell J.V.; Sulser F. | A possible common mechanism of action of antidepressant treatments - Reduction in the sensitivity of the noradrenergic cyclic AMP generating system in the rat limbic forebrain | 1976 | 293 | 2 | 109 | 114 | 351 |
| **16** | Engel G.; Hoyer D.; Berthold R.; Wagner H. | (±)[125Iodo]cyanopindolol, a new ligand for β-adrenoceptors: Identification and quantitation of subclasses of β-adrenoceptors in guinea pig | 1981 | 317 | 4 | 277 | 285 | 338 |
| **17** | Chaput Y.; de Montigny C.; Blier P. | Effects of a selective 5-HT reuptake blocker, citalopram, on the sensitivity of 5-HT autoreceptors: Electrophysiological studies in the rat brain | 1986 | 333 | 4 | 342 | 348 | 320 |
| **18** | Schlicker E.; Betz R.; Göthert M. | Histamine H3 receptor-mediated inhibition of serotonin release in the rat brain cortex | 1988 | 337 | 5 | 588 | 590 | 320 |
| **19** | Walters J.R.; Roth R.H. | Dopaminergic neurons: An in vivo system for measuring drug interactions with presynaptic receptors | 1976 | 296 | 1 | 5 | 14 | 311 |
| **20** | Hjorth S.; Magnusson T. | The 5-HT1A receptor agonist, 8-OH-DPAT, preferentially activates cell body 5-HT autoreceptors in rat brain in vivo | 1988 | 338 | 5 | 463 | 471 | 305 |
| **21** | Dubocovich M.L.; Masana M.I.; Iacob S.; Sauri D.M. | Melatonin receptor antagonists that differentiate hetween the human Mel(1a) and Mel(1b) recombinant subtypes are used to assess the pharmacological profile of the rabbit retina ML(1) presynaptic heteroreceptor | 1997 | 355 | 3 | 365 | 375 | 304 |
| **22** | Kathmann M.; Flau K.; Redmer A.; Tränkle C.; Schlicker E. | Cannabidiol is an allosteric modulator at mu- and delta-opioid receptors | 2006 | 372 | 5 | 354 | 361 | 304 |
| **23** | Strömbom U. | Catecholamine receptor agonists - Effects on motor activity and rate of tyrosine hydroxylation in mouse brain | 1976 | 292 | 2 | 167 | 176 | 303 |
| **24** | Hoffmann C.; Leitz M.R.; Oberdorf-Maass S.; Lohse M.J.; Klotz K.-N. | Comparative pharmacology of human β-adrenergic receptor subtypes - Characterization of stably transfected receptors in CHO cells | 2004 | 369 | 2 | 151 | 159 | 303 |
| **25** | Dumuis A.; Sebben M.; Bockaert J. | The gastrointestinal prokinetic benzamide derivatives are agonists at the non-classical 5-HT receptor (5-HT4) positively coupled to adenylate cyclase in neurons | 1989 | 340 | 4 | 403 | 410 | 298 |
| **26** | Gamse R.; Leeman S.E.; Holzer P.; Lembeck F. | Differential effects of capsaicin on the content of somatostatin, substance P, and neurotensin in the nervous system of the rat | 1981 | 317 | 2 | 140 | 148 | 296 |
| **27** | Lee C.-M.; Iversen L.L.; Hanley M.R.; Sandberg B.E.B. | The possible existence of multiple receptors for substance P | 1982 | 318 | 4 | 281 | 287 | 293 |
| **28** | Taube H.D.; Starke K.; Borowski E. | Presynaptic receptor systems on the noradrenergic neurones of rat brain | 1977 | 299 | 2 | 123 | 141 | 291 |
| **29** | Carlsson A.; Lindqvist M. | Dependence of 5-HT and catecholamine synthesis on concentrations of precursor amino-acids in rat brain | 1978 | 303 | 2 | 157 | 164 | 290 |
| **30** | Polc P.; Bonetti E.P.; Schaffner R.; Haefely W. | A three-state model of the benzodiazepine receptor explains the interactions between the benzodiazepine antagonist Ro 15-1788, benzodiazepine tranquilizers, β-carbolines, and phenobarbitone | 1982 | 321 | 4 | 260 | 264 | 290 |
| **31** | Miilsch A.; Busse R. | NG-nitro-L-arginine (N5-[imino(nitroamino)methyl]-L-ornithine) impairs endothelium-dependent dilations by inhibiting cytosolic nitric oxide synthesis from l-arginine | 1990 | 341 | 2-Jan | 143 | 147 | 286 |
| **32** | Starke K.; Montel H.; Gayk W.; Merker R. | Comparison of the effects of clonidine on pre-and postsynaptic adrenoceptors in the rabbit pulmonary artery - α-sympathomimetic inhibition of neurogenic vasoconstriction | 1974 | 285 | 2 | 133 | 150 | 285 |
| **33** | Gillen C.; Haurand M.; Kobelt D.J.; Wnendt S. | Affinity, potency and efficacy of tramadol and its metabolites at the cloned human μ-opioid receptor | 2000 | 362 | 2 | 116 | 121 | 273 |
| **34** | Kukovetz W.R.; Holzmann S.; Wurm A.; Pöch G. | Evidence for cyclic GMP-mediated relaxant effects of nitro-compounds in coronary smooth muscle | 1979 | 310 | 2 | 129 | 138 | 268 |
| **35** | Weitzell R.; Tanaka T.; Starke K. | Pre- and postsynaptic effects of yohimbine stereoisomers on noradrenergic transmission in the pulmonary artery of the rabbit | 1979 | 308 | 2 | 127 | 136 | 264 |
| **36** | Schwabe U.; Trost T. | Characterization of adenosine receptors in rat brain by (-)[3H]N6-phenylisopropyladenosine | 1980 | 313 | 3 | 179 | 187 | 262 |
| **37** | Schlicker E.; Fink K.; Hinterthaner M.; Göthert M. | Inhibition of noradrenaline release in the rat brain cortex via presynaptic H3 receptors | 1989 | 340 | 6 | 633 | 638 | 255 |
| **38** | Adell A.; Artigas F. | Differential effects of clomipramine given locally or systemically on extracellular 5-hydroxytryptamine in raphe nuclei and frontal cortex - An in vivo brain microdialysis study | 1991 | 343 | 3 | 237 | 244 | 255 |
| **39** | Costa M.; Furness J.B. | The peristaltic reflex: An analysis of the nerve pathways and their pharmacology | 1976 | 294 | 1 | 47 | 60 | 253 |
| **40** | Kehr W.; Carlsson A.; Lindqvist M. | A method for the determination of 3,4-dihydroxyphenylalanine (DOPA) in brain | 1972 | 274 | 3 | 273 | 280 | 251 |
| **41** | Klapproth H.; Reinheimer T.; Metzen J.; Münch M.; Bittinger F.; Kirkpatrick C.J.; Höhle K.-D.; Schemann M.; Racké K.; Wessler I. | Non-neuronal acetylcholine, a signalling molecule synthezised by surface cells of rat and man | 1997 | 355 | 4 | 515 | 523 | 248 |
| **42** | Fang J.; Bourin M.; Baker G.B. | Metabolism of risperidone to 9-hydroxyrisperidone by human cytochromes P450 2D6 and 3A4 | 1999 | 359 | 2 | 147 | 151 | 241 |
| **43** | Pauli-Magnus C.; Rekersbrink S.; Klotz U.; Fromm M.F. | Interaction of omeprazole, lansoprazole and pantoprazole with P-glycoprotein | 2001 | 364 | 6 | 551 | 557 | 235 |
| **44** | Hedqvist P.; Fredholm B.B. | Effects of adenosine on adrenergic neurotransmission; Prejunctional inhibition and postjunctional enhancement | 1976 | 293 | 3 | 217 | 223 | 234 |
| **45** | Starke K. | Alpha sympathomimetic inhibition of adrenergic and cholinergic transmission in the rabbit heart | 1972 | 274 | 1 | 18 | 45 | 232 |
| **46** | Westerink B.H.C.; Tuntler J.; Damsma G.; Rollema H.; de Vries J.B. | The use of tetrodotoxin for the characterization of drug-enhanced dopamine release in conscious rats studied by brain dialysis | 1987 | 336 | 5 | 502 | 507 | 232 |
| **47** | Le Poul E.; Laaris N.; Doucet E.; Laporte A.-M.; Hamon M.; Lanfumey L. | Early desensitization of somato-dendritic 5-HT1A autoreceptors in rats treated with fluoxetine or paroxetine | 1995 | 352 | 2 | 141 | 148 | 232 |
| **48** | Bruinvels A.T.; Palacios J.M.; Hoyer D. | Autoradiographic characterisation and localisation of 5-HT1D compared to 5-HT1B binding sites in rat brain | 1993 | 347 | 6 | 569 | 582 | 231 |
| **49** | Audinot V.; Mailliet F.; Lahaye-Brasseur C.; Bonnaud A.; Le Gall A.; Amossé C.; Dromaint S.; Rodriguez M.; Nagel N.; Galizzi J.-P.; Malpaux B.; Guillaumet G.; Lesieur D.; Lefoulon F.; Renard P.; Delagrange P.; Boutin J.A. | New selective ligands of human cloned melatonin MT1 and MT2 receptors | 2003 | 367 | 6 | 553 | 561 | 230 |
| **50** | Wiegand H.; Erdmann G.; Wellhöner H.H. | 125I-Labelled botulinum a neurotoxin: Pharmacokinetics in cats after intramuscular injection | 1976 | 292 | 2 | 161 | 165 | 224 |
| **51** | Kukovetz W.R.; Pöch G. | Inhibition of cyclic-3′,5′-nucleotide-phosphodiesterase as a possible mode of action of papaverine and similarly acting drugs | 1970 | 267 | 2 | 189 | 194 | 218 |
| **52** | Carlsson E.; Dahlöf C.-G.; Hedberg A.; Persson H.; Tångstrand B. | Differentiation of cardiac chronotropic and inotropic effects of β-adrenoceptor agonists | 1977 | 300 | 2 | 101 | 105 | 217 |
| **53** | Baxter G.S.; Craig D.A.; Clarke D.E. | 5-Hydroxytryptamine4 receptors mediate relaxation of the rat oesophageal tunica muscularis mucosae | 1991 | 343 | 5 | 439 | 446 | 212 |
| **54** | Costa B.; Colleoni M.; Conti S.; Parolaro D.; Franke C.; Trovato A.E.; Giagnoni G. | Oral anti-inflammatory activity of cannabidiol, a non-psychoactive constituent of cannabis, in acute carrageenan-induced inflammation in the rat paw | 2004 | 369 | 3 | 294 | 299 | 211 |
| **55** | O'Carroll A.-M.; Fowler C.J.; Phillips J.P.; Tobbia I.; Tipton K.F. | The deamination of dopamine by human brain monoamine oxidase - Specificity for the two enzyme forms in seven brain regions | 1983 | 322 | 3 | 198 | 202 | 210 |
| **56** | Samanin R.; Mennini T.; Ferraris A.; Bendotti C.; Borsini F.; Garattini S. | m-Chlorophenylpiperazine: A central serotonin agonist causing powerful anorexia in rats | 1979 | 308 | 2 | 159 | 163 | 208 |
| **57** | Starke K.; Reimann W.; Zumstein A.; Hertting G. | Effect of dopamine receptor agonists and antagonists on release of dopamine in the rabbit caudate nucleus in vitro | 1978 | 305 | 1 | 27 | 36 | 207 |
| **58** | Schoeffter P.; Hoyer D. | Interaction of arylpiperazines with 5-HT1A, 5-HT1B, 5-HT1C and 5-HT1D receptors: do discriminatory 5-HT1B receptor ligands exist? | 1989 | 339 | 6 | 675 | 683 | 206 |
| **59** | Knight A.R.; Misra A.; Quirk K.; Benwell K.; Revell D.; Kennett G.; Bickerdike M. | Pharmacological characterisation of the agonist radioligand binding site of 5-HT2A, 5-HT2B and 5-HT2C receptors | 2004 | 370 | 2 | 114 | 123 | 206 |
| **60** | Polc P.; Möhler H.; Haefely W. | The effect of diazepam on spinal cord activities: Possible sites and mechanisms of action | 1974 | 284 | 4 | 319 | 337 | 205 |
| **61** | Docherty J.R.; McGrath J.C. | A comparison of pre- and post-junctional potencies of several alpha-adrenoceptor agonists in the cardiovascular system and anococcygeus muscle of the rat - Evidence for two types of post-junctional alpha-adrenoceptor | 1980 | 312 | 2 | 107 | 116 | 205 |
| **62** | Zünkler B.J.; Lenzen S.; Manner K.; Panten U.; Trube G. | Concentration-dependent effects of tolbutamide, meglitinide, glipizide, glibenclamide and diazoxide on ATP-regulated K+ currents in pancreatic B-cells | 1988 | 337 | 2 | 225 | 230 | 204 |
| **63** | Gamse R. | Capsaicin and nociception in the rat and mouse - Possible role of substance P | 1982 | 320 | 3 | 205 | 216 | 202 |
| **64** | Schrör K.; Darius H.; Matzky R.; Ohlendorf R. | The antiplatelet and cardiovascular actions of a new carbacyclin derivative (ZK 36 374) - Equipotent to PGI2 in vitro | 1981 | 316 | 3 | 252 | 255 | 201 |
| **65** | Kroemer H.K.; Gautier J.-C.; Beaune P.; Henderson C.; Roland Wolf C.; Eichelbaum M. | Identification of P450 enzymes involved in metabolism of verapamil in humans | 1993 | 348 | 3 | 332 | 337 | 199 |
| **66** | Buchheit K.-H.; Engel G.; Mutschler E.; Richardson B. | Study of the contractile effect of 5-hydroxytryptamine (5-HT) in the isolated longitudinal muscle strip from guinea-pig ileum - Evidence for two distinct release mechanisms | 1985 | 329 | 1 | 36 | 41 | 198 |
| **67** | Baumann P.A.; Maître L. | Blockade of presynaptic α-receptors and of amine uptake in the rat brain by the antidepressant mianserine | 1977 | 300 | 1 | 31 | 37 | 193 |
| **68** | Pax R.; Bennett J.L.; Fetterer R. | A benzodiazepine derivative and praziquantel: Effects on musculature of Schistosoma mansoni and Schistosoma japonicum | 1978 | 304 | 3 | 309 | 315 | 192 |
| **69** | Markstein R.; Hoyer D.; Engel G. | 5-HT1A-receptors mediate stimulation of adenylate cyclase in rat hippocampus | 1986 | 333 | 4 | 335 | 341 | 192 |
| **70** | Mennini T.; Mocaer E.; Garattini S. | Tianeptine, a selective enhancer of serotonin uptake in rat brain | 1987 | 336 | 5 | 478 | 482 | 190 |
| **71** | Szolcsányi J.; Jancsó-Gábor A.; Joó F. | Functional and fine structural characteristics of the sensory neuron blocking effect of capsaicin | 1975 | 287 | 2 | 157 | 169 | 189 |
| **72** | Ikeda K.; Kobayashi S.; Suzuki M.; Miyata K.; Takeuchi M.; Yamada T.; Honda K. | M3 receptor antagonism by the novel antimuscarinic agent solifenacin in the urinary bladder and salivary gland | 2002 | 366 | 2 | 97 | 103 | 189 |
| **73** | Terranova J.-P.; Michaud J.-C.; Le Fur G.; Soubrié P. | Inhibition of long-term potentiation in rat hippocampal slices by anandamide and WIN55212-2: reversal by SR141716 A, a selective antagonist of CB1 cannabinoid receptors | 1995 | 352 | 5 | 576 | 579 | 188 |
| **74** | Westerink B.H.C.; Hofsteede H.M.; Damsma G.; de Vries J.B. | The significance of extracellular calcium for the release of dopamine, acetylcholine and amino acids in conscious rats, evaluated by brain microdialysis | 1988 | 337 | 4 | 373 | 378 | 186 |
| **75** | Nakazi M.; Bauer U.; Nickel T.; Kathmann M.; Schlicker E. | Inhibition of serotonin release in the mouse brain via presynaptic cannabinoid CB1 receptors | 2000 | 361 | 1 | 19 | 24 | 177 |
| **76** | Martin L.L.; Sanders-Bush E. | Comparison of the pharmacological characteristics of 5 HT1 and 5 HT2 binding sites with those of serotonin autoreceptors which modulate serotonin release | 1982 | 321 | 3 | 165 | 170 | 175 |
| **77** | Andén N.-E.; Golembiowska-Nikitin K.; Thornström U. | Selective stimulation of dopamine and noradrenaline autoreceptors by B-HT 920 and B-HT 933, respectively | 1982 | 321 | 2 | 100 | 104 | 174 |
| **78** | Engel G.; Göthert M.; Müller-Schweinitzer E.; Schlicker E.; Sistonen L.; Stadler P.A. | Evidence for common pharmacological properties of [3H]5-hydroxytryptamine binding sites, presynaptic 5-hydroxytryptamine autoreceptors in CNS and inhibitory presynaptic 5-hydroxytryptamine receptors on sympathetic nerves | 1983 | 324 | 2 | 116 | 124 | 174 |
| **79** | López-Giménez J.F.; Mengod G.; Palacios J.M.; Vilaró M.T. | Selective visualization of rat brain 5-HT(2A) receptors by autoradiography with [3H]MDL 100,907 | 1997 | 356 | 4 | 446 | 454 | 173 |
| **80** | Posadas I.; Terencio M.C.; Guillén I.; Ferrándiz M.L.; Coloma J.; Payá M.; Alcaraz M.J. | Co-regulation between cyclo-oxygenase-2 and inducible nitric oxide synthase expression in the time-course of murine inflammation | 2000 | 361 | 1 | 98 | 106 | 171 |
| **81** | Haeusler G. | Clonidine-induced inhibition of sympathetic nerve activity: No indication for a central presynaptic or an indirect sympathomimetic mode of action | 1974 | 286 | 1 | 97 | 111 | 170 |
| **82** | Dompert W.U.; Glaser T.; Traber J. | 3H-TVX Q 7821: identification of 5-HT1 binding sites as target for a novel putative anxiolytic | 1985 | 328 | 4 | 467 | 470 | 170 |
| **83** | Blier P.; Chaput Y.; de Montigny C. | Long-term 5-HT reuptake blockade, but not monoamine oxidase inhibition, decreases the function of terminal 5-HT autoreceptors: an electrophysiological study in the rat brain | 1988 | 337 | 3 | 246 | 254 | 170 |
| **84** | Andén N.-E.; Stock G. | Inhibitory effect of gammahydroxybutyric acid and gammaaminobutyric acid on the dopamine cells in the substantia nigra | 1973 | 279 | 1 | 89 | 92 | 167 |
| **85** | Waeber C.; Schoeffter P.; Palacios J.M.; Hoyer D. | Molecular pharmacology of 5-HT1D recognition sites: Radioligand binding studies in human, pig and calf brain membranes | 1988 | 337 | 6 | 595 | 601 | 167 |
| **86** | Santiago M.; Westerink B.H.C. | Characterization of the in vivo release of dopamine as recorded by different types of intracerebral microdialysis probes | 1990 | 342 | 4 | 407 | 414 | 167 |
| **87** | Atack C.; Lindqvist M. | Conjoint native and orthophthaldialdehyde-condensate assays for the fluorimetric determination of 5-hydroxyindoles in brain | 1973 | 279 | 3 | 267 | 284 | 166 |
| **88** | Cunha R.A.; Johansson B.; Constantino M.D.; Sebastião A.M.; Fredholm B.B. | Evidence for high-affinity binding sites for the adenosine A2A receptor agonist [3H] CGS 21680 in the rat hippocampus and cerebral cortex that are different from striatal A2A receptors | 1996 | 353 | 3 | 261 | 271 | 166 |
| **89** | Ongini E.; Dionisotti S.; Gessi S.; Irenius E.; Fredholm B.B. | Comparison of CGS 15943, ZM 241385 and SCH 58261 as antagonists at human adenosine receptors | 1999 | 359 | 1 | 7 | 10 | 166 |
| **90** | Timmermans P.B.M.W.M.; Kwa H.Y.; van Zwieten P.A. | Possible subdivision of postsynaptic α-adrenoceptors mediating pressor responses in the pithed rat | 1979 | 310 | 2 | 189 | 193 | 164 |
| **91** | Lohse M.J.; Klotz K.-N.; Schwabe U.; Cristalli G.; Vittori S.; Grifantini M. | 2-Chloro-N6-cyclopentyladenosine: a highly selective agonist at A1 adenosine receptors | 1988 | 337 | 6 | 687 | 689 | 164 |
| **92** | Bayer R.; Kalusche D.; Kaufmann R.; Mannhold R. | Inotropic and electrophysiological actions of verapamil and D 600 in mammalian myocardium - III. Effects of the optical isomers on transmembrane action potentials | 1975 | 290 | 1 | 81 | 97 | 163 |
| **93** | Langer S.Z.; Briley M.S.; Raisman R.; Henry J.-F.; Morselli P.L. | Specific 3H-imipramine binding in human platelets - Influence of age and sex | 1980 | 313 | 3 | 189 | 194 | 163 |
| **94** | Arbilla S.; Depoortere H.; George P.; Langer S.Z. | Pharmacological profile of the imidazopyridine zolpidem at benzodiazepine receptors and electrocorticogram in rats | 1985 | 330 | 3 | 248 | 251 | 163 |
| **95** | Dunwiddie T.V.; Hoffer B.J.; Fredholm B.B. | Alkylxanthines elevate hippocampal excitability - Evidence for a role of endogenous adenosine | 1981 | 316 | 4 | 326 | 330 | 162 |
| **96** | Doxey J.C.; Lane A.C.; Roach A.G.; Virdce N.K. | Comparison of the α-adrenoceptor antagonist profiles of idazoxan (RX 781094), yohimbine, rauwolscine and corynanthine | 1984 | 325 | 2 | 136 | 144 | 161 |
| **97** | Rübsamen K.; Breithaupt H.; Habermann B. | Biochemistry and pharmacology of the crotoxin complex - I. Subfractionation and recombination of the crotoxin complex | 1971 | 270 | 3 | 274 | 288 | 160 |
| **98** | Schoemaker H.; Pimoule C.; Arbilla S.; Scatton B.; Javoy-Agid F.; Langer S.Z. | Sodium dependent [3H]cocaine binding associated with dopamine uptake sites in the rat striatum and human putamen decrease after dopaminergic denervation and in Parkinsons disease | 1985 | 329 | 3 | 227 | 235 | 160 |
| **99** | Kohlhardt M.; Fleckenstein A. | Inhibition of the slow inward current by nifedipine in mammalian ventricular myocardium | 1977 | 298 | 3 | 267 | 272 | 159 |
| **100** | Busse R.; Lackhoff A.; Bassenge E. | Endothelium-derived relaxant factor inhibits platelet activation | 1987 | 336 | 5 | 566 | 571 | 158 |

**Supplementary Table 1:** The list of **top 100 most cited research articles** in Naunyn-Schmiedeberg’s Archives of Pharmacology (1969–2024), ranked by total citation count.

| **S#** | **Author** | **Total Citations** | **Number of Publications** | **h_index** | **g_index** | **m_index** | **HG -Composite** | **Q2 Index** | **Publication Year Start** |
| --- | --- | --- | --- | --- | --- | --- | --- | --- | --- |
| 1 | SCHLICKER E | 1870 | 6 | 6 | 6 | 0.14 | 6 | 0.92 | 1983 |
| 2 | HOYER D | 1774 | 6 | 6 | 6 | 0.133 | 6 | 0.89 | 1981 |
| 3 | LEMBECK F | 1721 | 3 | 3 | 3 | 0.064 | 3 | 0.44 | 1979 |
| 4 | STARKE K | 1694 | 6 | 6 | 6 | 0.111 | 6 | 0.82 | 1972 |
| 5 | ENGEL G | 1542 | 5 | 5 | 5 | 0.111 | 5 | 0.74 | 1981 |
| 6 | GÖTHERT M | 1389 | 4 | 4 | 4 | 0.093 | 4 | 0.61 | 1983 |
| 7 | HOLZER P | 1353 | 2 | 2 | 2 | 0.043 | 2 | 0.29 | 1979 |
| 8 | KLOTZ K-N | 1342 | 4 | 4 | 4 | 0.103 | 4 | 0.64 | 1987 |
| 9 | LOHSE MJ | 1342 | 4 | 4 | 4 | 0.103 | 4 | 0.64 | 1987 |
| 10 | LINDQVIST M | 1311 | 4 | 4 | 4 | 0.074 | 4 | 0.54 | 1972 |
| 11 | FREDHOLM BB | 1214 | 5 | 5 | 5 | 0.1 | 5 | 0.71 | 1976 |
| 12 | CARLSSON A | 1145 | 3 | 3 | 3 | 0.056 | 3 | 0.41 | 1972 |
| 13 | KEHR W | 855 | 2 | 2 | 2 | 0.037 | 2 | 0.27 | 1972 |
| 14 | HANDLEY SL | 820 | 1 | 1 | 1 | 0.024 | 1 | 0.15 | 1984 |
| 15 | MITHANI S | 820 | 1 | 1 | 1 | 0.024 | 1 | 0.15 | 1984 |
| 16 | SCHWABE U | 815 | 3 | 3 | 3 | 0.065 | 3 | 0.44 | 1980 |
| 17 | TAUBE HD | 706 | 2 | 2 | 2 | 0.039 | 2 | 0.28 | 1975 |
| 18 | ANDÉN N-E | 699 | 3 | 3 | 3 | 0.057 | 3 | 0.41 | 1973 |
| 19 | STRÖMBOM U | 661 | 2 | 2 | 2 | 0.04 | 2 | 0.28 | 1976 |
| 20 | HILLENBRAND K | 640 | 1 | 1 | 1 | 0.025 | 1 | 0.16 | 1986 |
| 21 | ATACK CV | 604 | 1 | 1 | 1 | 0.019 | 1 | 0.14 | 1972 |
| 22 | DAVIS JN | 604 | 1 | 1 | 1 | 0.019 | 1 | 0.14 | 1972 |
| 23 | WESTERINK BHC | 585 | 3 | 3 | 3 | 0.077 | 3 | 0.48 | 1987 |
| 24 | PALACIOS JM | 571 | 3 | 3 | 3 | 0.079 | 3 | 0.49 | 1988 |
| 25 | GAMSE R | 498 | 2 | 2 | 2 | 0.044 | 2 | 0.30 | 1981 |
| 26 | HAEFELY W | 495 | 2 | 2 | 2 | 0.038 | 2 | 0.28 | 1974 |
| 27 | POLC P | 495 | 2 | 2 | 2 | 0.038 | 2 | 0.28 | 1974 |
| 28 | BLIER P | 490 | 2 | 2 | 2 | 0.05 | 2 | 0.32 | 1986 |
| 29 | CHAPUT Y | 490 | 2 | 2 | 2 | 0.05 | 2 | 0.32 | 1986 |
| 30 | DE MONTIGNY C | 490 | 2 | 2 | 2 | 0.05 | 2 | 0.32 | 1986 |
| 31 | LANGER SZ | 486 | 3 | 3 | 3 | 0.065 | 3 | 0.44 | 1980 |
| 32 | KUKOVETZ WR | 486 | 2 | 2 | 2 | 0.036 | 2 | 0.27 | 1970 |
| 33 | PÖCH G | 486 | 2 | 2 | 2 | 0.036 | 2 | 0.27 | 1970 |
| 34 | HEGLER J | 486 | 1 | 1 | 1 | 0.034 | 1 | 0.18 | 1997 |
| 35 | HESSLING J | 486 | 1 | 1 | 1 | 0.034 | 1 | 0.18 | 1997 |
| 36 | KULL B | 486 | 1 | 1 | 1 | 0.034 | 1 | 0.18 | 1997 |
| 37 | OWMAN C | 486 | 1 | 1 | 1 | 0.034 | 1 | 0.18 | 1997 |
| 38 | KATHMANN M | 481 | 2 | 2 | 2 | 0.077 | 2 | 0.39 | 2000 |
| 39 | KOSTERLITZ HW | 450 | 1 | 1 | 1 | 0.023 | 1 | 0.15 | 1982 |
| 40 | MAGNAN J | 450 | 1 | 1 | 1 | 0.023 | 1 | 0.15 | 1982 |
| 41 | PATERSON SJ | 450 | 1 | 1 | 1 | 0.023 | 1 | 0.15 | 1982 |
| 42 | TAVANI A | 450 | 1 | 1 | 1 | 0.023 | 1 | 0.15 | 1982 |
| 43 | BUSSE R | 444 | 2 | 2 | 2 | 0.051 | 2 | 0.32 | 1987 |
| 44 | AGHAJANIAN GK | 427 | 1 | 1 | 1 | 0.02 | 1 | 0.14 | 1977 |
| 45 | BUNNEY BS | 427 | 1 | 1 | 1 | 0.02 | 1 | 0.14 | 1977 |
| 46 | FÖRSTERMANN U | 426 | 1 | 1 | 1 | 0.032 | 1 | 0.18 | 1995 |
| 47 | KLEINERT H | 426 | 1 | 1 | 1 | 0.032 | 1 | 0.18 | 1995 |
| 48 | DAMSMA G | 418 | 2 | 2 | 2 | 0.051 | 2 | 0.32 | 1987 |
| 49 | DE VRIES JB | 418 | 2 | 2 | 2 | 0.051 | 2 | 0.32 | 1987 |
| 50 | ENDO T | 415 | 1 | 1 | 1 | 0.02 | 1 | 0.14 | 1975 |

**Supplementary Table 2:** The 50 most productive authors in the **top 100 most cited research articles** of *Naunyn-Schmiedeberg’s Archives of Pharmacology* (1969–2024), ranked based on total citations.

| **S#** | Authors | Title | Year | Volume | Issue | Page start | Page end | Cited by |
| --- | --- | --- | --- | --- | --- | --- | --- | --- |
| 1 | Zimmermann H. | Extracellular metabolism of ATP and other nucleotides | 2000 | 362 | 5-Apr | 299 | 309 | 862 |
| 2 | Zanger U.M.; Raimundo S.; Eichelbaum M. | Cytochrome P450 2D6: Overview and update on pharmacology, genetics, biochemistry | 2004 | 369 | 1 | 23 | 37 | 751 |
| 3 | Seifert R.; Wenzel-Seifert K. | Constitutive activity of G-proteins-coupled receptors: Cause of disease and common property of wild-type receptors | 2002 | 366 | 5 | 381 | 416 | 545 |
| 4 | Fredholm B.B.; Arslan G.; Halldner L.; Kull B.; Schulte G.; Wasserman W. | Structure and function of adenosine receptors and their genes | 2000 | 362 | 5-Apr | 364 | 374 | 529 |
| 5 | Von Kugelgen I.; Wetter A. | Molecular pharmacology of P2Y-receptors | 2000 | 362 | 5-Apr | 310 | 323 | 446 |
| 6 | Feelisch M. | The use of nitric oxide donors in pharmacological studies | 1998 | 358 | 1 | 113 | 122 | 320 |
| 7 | König J.; Seithel A.; Gradhand U.; Fromm M.F. | Pharmacogenomics of human OATP transporters | 2006 | 372 | 6 | 432 | 443 | 318 |
| 8 | Piechota-Polanczyk A.; Fichna J. | Review article: The role of oxidative stress in pathogenesis and treatment of inflammatory bowel diseases | 2014 | 387 | 7 | 605 | 620 | 317 |
| 9 | Klotz K.-N. | Adenosine receptors and their ligands | 2000 | 362 | 5-Apr | 382 | 391 | 308 |
| 10 | Ingelman-Sundberg M. | Human drug metabolising cytochrome P450 enzymes: Properties and polymorphisms | 2004 | 369 | 1 | 89 | 104 | 303 |
| 11 | Michel M.C.; Wieland T.; Tsujimoto G. | How reliable are G-protein-coupled receptor antibodies? | 2009 | 379 | 4 | 385 | 388 | 267 |
| 12 | Alemany R.; Van Koppen C.J.; Danneberg K.; Ter Braak M.; Meyer Zu Heringdorf D. | Regulation and functional roles of sphingosine kinases | 2007 | 374 | 6-May | 413 | 428 | 217 |
| 13 | Norenberg W.; Illes P. | Neuronal P2X receptors: Localisation and functional properties | 2000 | 362 | 5-Apr | 324 | 339 | 205 |
| 14 | Hoyer D.; Lübbert H.; Bruns C. | Molecular pharmacology of somatostatin receptors | 1994 | 350 | 5 | 441 | 453 | 205 |
| 15 | Haas H.L.; Selbach O. | Functions of neuronal adenosine receptors | 2000 | 362 | 5-Apr | 375 | 381 | 188 |
| 16 | Chatterjee P.K. | Novel pharmacological approaches to the treatment of renal ischemia-reperfusion injury: A comprehensive review | 2007 | 376 | 2-Jan | 1 | 43 | 188 |
| 17 | Michel M.C.; Kenny B.; Schwinn D.A. | Classification of α1-adrenoceptor subtypes | 1995 | 352 | 1 | 1 | 10 | 188 |
| 18 | Gawel K.; Gibula E.; Marszalek-Grabska M.; Filarowska J.; Kotlinska J.H. | Assessment of spatial learning and memory in the Barnes maze task in rodents—methodological consideration | 2019 | 392 | 1 |  |  | 174 |
| 19 | Kaumann A.J.; Molenaar P. | Modulation of human cardiac function through 4 β-adrenoceptor populations | 1997 | 355 | 6 | 667 | 681 | 172 |
| 20 | Villalón C.M.; Centurión D. | Cardiovascular responses produced by 5-hydroxytriptamine: A pharmacological update on the receptors/mechanisms involved and therapeutic implications | 2007 | 376 | 2-Jan | 45 | 63 | 166 |
| 21 | Lambrecht G. | Agonists and antagonists acting at P2X receptors: Selectivity profiles and functional implications | 2000 | 362 | 5-Apr | 340 | 350 | 164 |
| 22 | Avelino A.; Cruz F. | TRPV1 (vanilloid receptor) in the urinary tract: Expression, function and clinical applications | 2006 | 373 | 4 | 287 | 299 | 159 |
| 23 | Geyer J.; Wilke T.; Petzinger E. | The solute carrier family SLC10: More than a family of bile acid transporters regarding function and phylogenetic relationships | 2006 | 372 | 6 | 413 | 431 | 153 |
| 24 | Lovinger D.M. | Alcohols and neurotransmitter gated ion channels: Past, present and future | 1997 | 356 | 3 | 267 | 282 | 150 |
| 25 | Bedi O.; Dhawan V.; Sharma P.L.; Kumar P. | Pleiotropic effects of statins: new therapeutic targets in drug design | 2016 | 389 | 7 | 695 | 712 | 141 |
| 26 | Harteneck C. | Function and pharmacology of TRPM cation channels | 2005 | 371 | 4 | 307 | 314 | 140 |
| 27 | Denac H.; Mevissen M.; Scholtysik G. | Structure, function and pharmacology of voltage-gated sodium channels | 2000 | 362 | 6 | 453 | 479 | 138 |
| 28 | Chen J.; Hackos D.H. | TRPA1 as a drug target - Promise and challenges | 2015 | 388 | 4 | 451 | 463 | 137 |
| 29 | Oswald C.; Holland I.B.; Schmitt L. | The motor domains of ABC-transporters: What can structures tell us? | 2006 | 372 | 6 | 385 | 399 | 129 |
| 30 | Smolenski A.; Burkhardt A.M.; Eigenthaler M.; Butt E.; Gambaryan S.; Lohmann S.M.; Walter U. | Functional analysis of cGMP-dependent protein kinases I and II as mediators of NO/cGMP effects | 1998 | 358 | 1 | 134 | 139 | 129 |
| 31 | Mayer B.; Werner E.R. | In search of a function for tetrahydrobiopterin in the biosynthesis of nitric oxide | 1995 | 351 | 5 | 453 | 463 | 127 |
| 32 | Burk O.; Wojnowski L. | Cytochrome P450 3A and their regulation | 2004 | 369 | 1 | 105 | 124 | 125 |
| 33 | Leineweber K.; Büscher R.; Bruck H.; Brodde O.-E. | β-Adrenoceptor polymorphisms | 2004 | 369 | 1 | 1 | 22 | 124 |
| 34 | Glatt H.; Meinl W. | Pharmacogenetics of soluble sulfotransferases (SULTs) | 2004 | 369 | 1 | 55 | 68 | 123 |
| 35 | Gach K.; Wyrębska A.; Fichna J.; Janecka A. | The role of morphine in regulation of cancer cell growth | 2011 | 384 | 3 | 221 | 230 | 122 |
| 36 | Hassani F.V.; Shirani K.; Hosseinzadeh H. | Rosemary (Rosmarinus officinalis) as a potential therapeutic plant in metabolic syndrome: a review | 2016 | 389 | 9 | 931 | 949 | 121 |
| 37 | Maarsingh H.; Pera T.; Meurs H. | Arginase and pulmonary diseases | 2008 | 378 | 2 | 171 | 184 | 114 |
| 38 | Michel M.C.; Li Y.; Heusch G. | Mitogen-activated protein kinases in the heart | 2001 | 363 | 3 | 245 | 266 | 112 |
| 39 | Workman A.J. | Cardiac adrenergic control and atrial fibrillation | 2010 | 381 | 3 | 235 | 249 | 110 |
| 40 | Beattie D.T.; Smith J.A.M. | Serotonin pharmacology in the gastrointestinal tract: A review | 2008 | 377 | 3 | 181 | 203 | 102 |
| 41 | Vogelsgesang M.; Pautsch A.; Aktories K. | C3 exoenzymes, novel insights into structure and action of Rho-ADP-ribosylating toxins | 2007 | 374 | 6-May | 347 | 360 | 102 |
| 42 | Deussen A. | Metabolic flux rates of adenosine in the heart | 2000 | 362 | 5-Apr | 351 | 363 | 101 |
| 43 | Fozard J.R.; Kalkman H.O. | 5-Hydroxytryptamine (5-HT) and the initiation of migraine: new perspectives | 1994 | 350 | 3 | 225 | 229 | 100 |
| 44 | Friebe A.; Sandner P.; Schmidtko A. | cGMP: a unique 2nd messenger molecule – recent developments in cGMP research and development | 2020 | 393 | 2 | 287 | 302 | 99 |
| 45 | Oude Weernink P.A.; López De Jesús M.; Schmidt M. | Phospholipase D signaling: Orchestration by PIP2 and small GTPases | 2007 | 374 | 6-May | 399 | 411 | 95 |
| 46 | Modjtahedi H.; Cho B.C.; Michel M.C.; Solca F. | A comprehensive review of the preclinical efficacy profile of the ErbB family blocker afatinib in cancer | 2014 | 387 | 6 | 505 | 521 | 93 |
| 47 | Vrydag W.; Michel M.C. | Tools to study β3-adrenoceptors | 2007 | 374 | 6-May | 385 | 398 | 92 |
| 48 | Van Abel M.; Hoenderop J.G.J.; Bindels R.J.M. | The epithelial calcium channels TRPV5 and TRPV6: Regulation and implications for disease | 2005 | 371 | 4 | 295 | 306 | 92 |
| 49 | Arch J.R.S. | The discovery of drugs for obesity, the metabolic effects of leptin and variable receptor pharmacology: Perspectives from β3- adrenoceptor agonists | 2008 | 378 | 2 | 225 | 240 | 92 |
| 50 | Eichhorn B.; Dobrev D. | Vascular large conductance calcium-activated potassium channels: Functional role and therapeutic potential | 2007 | 376 | 3 | 145 | 155 | 88 |
| 51 | Dietrich A.; Mederos Y Schnitzler M.; Kalwa H.; Storch U.; Gudermann T. | Functional characterization and physiological relevance of the TRPC3/6/7 subfamily of cation channels | 2005 | 371 | 4 | 257 | 265 | 85 |
| 52 | Gross G.; Wicke K.; Drescher K.U. | Dopamine D3 receptor antagonism - Still a therapeutic option for the treatment of schizophrenia | 2013 | 386 | 2 | 155 | 166 | 84 |
| 53 | Petzinger E.; Geyer J. | Drug transporters in pharmacokinetics | 2006 | 372 | 6 | 465 | 475 | 84 |
| 54 | Mayer B.; Andrew P. | Nitric oxide synthases: Catalytic function and progress towards selective inhibition | 1998 | 358 | 1 | 127 | 133 | 81 |
| 55 | Gavioli E.C.; Calo' G. | Antidepressant- and anxiolytic-like effects of nociceptin/orphanin FQ receptor ligands | 2006 | 372 | 5 | 319 | 330 | 80 |
| 56 | Savelieva I.; Kourliouros A.; Camm J. | Primary and secondary prevention of atrial fibrillation with statins and polyunsaturated fatty acids: Review of evidence and clinical relevance | 2010 | 381 | 3 | 207 | 219 | 78 |
| 57 | Dhein S.; Hagen A.; Jozwiak J.; Dietze A.; Garbade J.; Barten M.; Kostelka M.; Mohr F.-W. | Improving cardiac gap junction communication as a new antiarrhythmic mechanism: The action of antiarrhythmic peptides | 2010 | 381 | 3 | 221 | 234 | 77 |
| 58 | Offermanns S.; Schultz G. | Complex information processing by the transmembrane signaling system involving G proteins | 1994 | 350 | 4 | 329 | 338 | 76 |
| 59 | Igawa Y.; Michel M.C. | Pharmacological profile of β3-adrenoceptor agonists in clinical development for the treatment of overactive bladder syndrome | 2013 | 386 | 3 | 177 | 183 | 73 |
| 60 | Campian M.E.; Hardziyenka M.; Michel M.C.; Tan H.L. | How valid are animal models to evaluate treatments for pulmonary hypertension? | 2006 | 373 | 6 | 391 | 400 | 73 |
| 61 | Ghasemzadeh Rahbardar M.; Hosseinzadeh H. | Effects of rosmarinic acid on nervous system disorders: an updated review | 2020 | 393 | 10 | 1779 | 1795 | 73 |
| 62 | McNulty A.L.; Leddy H.A.; Liedtke W.; Guilak F. | TRPV4 as a therapeutic target for joint diseases | 2015 | 388 | 4 | 437 | 450 | 72 |
| 63 | Andra S.; Balu S.K.; Jeevanandham J.; Muthalagu M.; Vidyavathy M.; Chan Y.S.; Danquah M.K. | Phytosynthesized metal oxide nanoparticles for pharmaceutical applications | 2019 | 392 | 7 | 755 | 771 | 72 |
| 64 | Vauquelin G.; Bostoen S.; Vanderheyden P.; Seeman P. | Clozapine, atypical antipsychotics, and the benefits of fast-off D 2 dopamine receptor antagonism | 2012 | 385 | 4 | 337 | 372 | 70 |
| 65 | Andra S.; Balu S.; Jeevanandam J.; Muthalagu M. | Emerging nanomaterials for antibacterial textile fabrication | 2021 | 394 | 7 | 1355 | 1382 | 70 |
| 66 | Anand P.; Singh B.; Jaggi A.S.; Singh N. | Mast cells: An expanding pathophysiological role from allergy to other disorders | 2012 | 385 | 7 | 657 | 670 | 69 |
| 67 | Plant T.D.; Schaefer M. | Receptor-operated cation channels formed by TRPC4 and TRPC5 | 2005 | 371 | 4 | 266 | 276 | 68 |
| 68 | Humphrey P.P.A.; Buell G.; Kennedy I.; Khakh B.S.; Michel A.D.; Surprenant A.; Trezise D.J. | New insights on P2X purinoceptors | 1995 | 352 | 6 | 585 | 596 | 67 |
| 69 | Bonvini S.J.; Birrell M.A.; Smith J.A.; Belvisi M.G. | Targeting TRP channels for chronic cough: From bench to bedside | 2015 | 388 | 4 | 401 | 420 | 67 |
| 70 | Gudermann T.; Grosse R.; Schultz G. | Contribution of receptor/G protein signaling to cell growth and transformation | 2000 | 361 | 4 | 345 | 362 | 66 |
| 71 | Kim H.A.; Miller A.A.; Drummond G.R.; Thrift A.G.; Arumugam T.V.; Phan T.G.; Srikanth V.K.; Sobey C.G. | Vascular cognitive impairment and Alzheimer's disease: Role of cerebral hypoperfusion and oxidative stress | 2012 | 385 | 10 | 953 | 959 | 65 |
| 72 | Wieland T.; Chen C.-K. | Regulators of G-protein signalling: A novel protein family involved in timely deactivation and desensitization of signalling via heterotrimeric G proteins | 1999 | 360 | 1 | 14 | 26 | 63 |
| 73 | Nishida M.; Kurose H. | Roles of TRP channels in the development of cardiac hypertrophy | 2008 | 378 | 4 | 395 | 406 | 61 |
| 74 | Batiha G.E.-S.; Teibo J.O.; Wasef L.; Shaheen H.M.; Akomolafe A.P.; Teibo T.K.A.; Al-kuraishy H.M.; Al-Garbeeb A.I.; Alexiou A.; Papadakis M. | A review of the bioactive components and pharmacological properties of Lavandula species | 2023 | 396 | 5 | 877 | 900 | 60 |
| 75 | Quast U. | ATP-sensitive K+ channels in the kidney | 1996 | 354 | 3 | 213 | 225 | 60 |
| 76 | Molderings G.J.; Haenisch B.; Brettner S.; Homann J.; Menzen M.; Dumoulin F.L.; Panse J.; Butterfield J.; Afrin L.B. | Pharmacological treatment options for mast cell activation disease | 2016 | 389 | 7 | 671 | 694 | 59 |
| 77 | Kryzhanovsky G.N. | The mechanism of action of tetanus toxin: Effect on synaptic processes and some particular features of toxin binding by the nervous tissue | 1973 | 276 | 4-Mar | 247 | 270 | 57 |
| 78 | Brodde O.-E. | β-adrenoceptor blocker treatment and the cardiac β-adrenoceptor- G-protein(s)-adenylyl cyclase system in chronic heart failure | 2007 | 374 | 6-May | 361 | 372 | 56 |
| 79 | Chai W.; Danser A.H.J. | Why are mineralocorticoid receptor antagonists cardioprotective? | 2006 | 374 | 3 | 153 | 162 | 55 |
| 80 | Imenshahidi M.; Karimi G.; Hosseinzadeh H. | Effects of melatonin on cardiovascular risk factors and metabolic syndrome: a comprehensive review | 2020 | 393 | 4 | 521 | 536 | 54 |
| 81 | Penn R.B. | Embracing emerging paradigms of G protein-coupled receptor agonism and signaling to address airway smooth muscle pathobiology in asthma | 2008 | 378 | 2 | 149 | 169 | 52 |
| 82 | Gerloff T. | Impact of genetic polymorphisms in transmembrane carrier-systems on drug and xenobiotic distribution | 2004 | 369 | 1 | 69 | 77 | 50 |
| 83 | Onohuean H.; Al-kuraishy H.M.; Al-Gareeb A.I.; Qusti S.; Alshammari E.M.; Batiha G.E.-S. | Covid-19 and development of heart failure: mystery and truth | 2021 | 394 | 10 | 2013 | 2021 | 50 |
| 84 | Hemmings D.G. | Signal transduction underlying the vascular effects of sphingosine 1-phosphate and sphingosylphosphorylcholine | 2006 | 373 | 1 | 18 | 29 | 49 |
| 85 | Hammond J.R. | Interaction of a series of draflazine analogues with equilibrative nucleoside transporters: Species differences and transporter subtype selectivity | 2000 | 361 | 4 | 373 | 382 | 49 |
| 86 | Motulsky H.J. | Common misconceptions about data analysis and statistics | 2014 | 387 | 11 | 1017 | 1023 | 48 |
| 87 | Attwood P.V.; Wieland T. | Nucleoside diphosphate kinase as protein histidine kinase | 2015 | 388 | 2 | 153 | 160 | 46 |
| 88 | Hamimed S.; Jabberi M.; Chatti A. | Nanotechnology in drug and gene delivery | 2022 | 395 | 7 | 769 | 787 | 46 |
| 89 | Koesling D. | Modulators of soluble guanylyl cyclase | 1998 | 358 | 1 | 123 | 126 | 46 |
| 90 | Franke H.; Scholl R.; Aigner A. | Ricin and Ricinus communis in pharmacology and toxicology-from ancient use and “Papyrus Ebers” to modern perspectives and “poisonous plant of the year 2018” | 2019 | 392 | 10 | 1181 | 1208 | 45 |
| 91 | Köles L.; Gerevich Z.; Oliveira J.F.; Zadori Z.S.; Wirkner K.; Illes P. | Interaction of P2 purinergic receptors with cellular macromolecules | 2008 | 377 | 1 | 1 | 33 | 44 |
| 92 | Tawfik M.; Chen F.; Goldberg J.L.; Sabel B.A. | Nanomedicine and drug delivery to the retina: current status and implications for gene therapy | 2022 | 395 | 12 | 1477 | 1507 | 44 |
| 93 | Kaumann A.J.; Lynham J.A.; Brown A.M. | Comparison of the densities of 5-HT4 receptors, β1- and β2-adrenoceptors in human atrium: Functional implications | 1996 | 353 | 5 | 592 | 595 | 44 |
| 94 | Wieland T. | Interaction of nucleoside diphosphate kinase B with heterotrimeric G protein βγ dimers: Consequences on G protein activation and stability | 2007 | 374 | 6-May | 373 | 383 | 43 |
| 95 | Marino N.; Marshall J.-C.; Steeg P.S. | Protein-protein interactions: A mechanism regulating the anti-metastatic properties of Nm23-H1 | 2011 | 384 | 5-Apr | 351 | 362 | 43 |
| 96 | Niemeyer B.A. | Structure-function analysis of TRPV channels | 2005 | 371 | 4 | 285 | 294 | 42 |
| 97 | Ostrom R.S.; Bogard A.S.; Gros R.; Feldman R.D. | Choreographing the adenylyl cyclase signalosome: Sorting out the partners and the steps | 2012 | 385 | 1 | 5 | 12 | 41 |
| 98 | Houshmandfar S.; Saeedi-Boroujeni A.; Rashno M.; Khodadadi A.; Mahmoudian-Sani M.-R. | miRNA-223 as a regulator of inflammation and NLRP3 inflammasome, the main fragments in the puzzle of immunopathogenesis of different inflammatory diseases and COVID-19 | 2021 | 394 | 11 | 2187 | 2195 | 41 |
| 99 | Mandal P. | Potential biomarkers associated with oxidative stress for risk assessment of colorectal cancer | 2017 | 390 | 6 | 557 | 565 | 41 |
| 100 | Batiha G.E.-S.; Wasef L.; Teibo J.O.; Shaheen H.M.; Zakariya A.M.; Akinfe O.A.; Teibo T.K.A.; Al-kuraishy H.M.; Al-Garbee A.I.; Alexiou A.; Papadakis M. | Commiphora myrrh: a phytochemical and pharmacological update | 2023 | 396 | 3 | 405 | 420 | 40 |

**Supplementary Table 3:** The list of **top 100 most cited reviews** in Naunyn-Schmiedeberg’s Archives of Pharmacology (1969–2024), ranked by total citation count.

| **S#** | **Author** | **Total Citations** | **Number of Publications** | **h_index** | **g_index** | **m_index** | **Hg Composite** | **Q2 Index** | **Publication Year Start** |
| --- | --- | --- | --- | --- | --- | --- | --- | --- | --- |
| 1 | MICHEL MC | 898 | 7 | 7 | 7 | 0.226 | 7 | 1.257776 | 1995 |
| 2 | ZIMMERMANN H | 862 | 1 | 1 | 1 | 0.038 | 1 | 0.194936 | 2000 |
| 3 | EICHELBAUM M | 751 | 1 | 1 | 1 | 0.045 | 1 | 0.212132 | 2004 |
| 4 | RAIMUNDO S | 751 | 1 | 1 | 1 | 0.045 | 1 | 0.212132 | 2004 |
| 5 | ZANGER UM | 751 | 1 | 1 | 1 | 0.045 | 1 | 0.212132 | 2004 |
| 6 | SEIFERT R | 545 | 1 | 1 | 1 | 0.042 | 1 | 0.204939 | 2002 |
| 7 | WENZEL-SEIFERT K | 545 | 1 | 1 | 1 | 0.042 | 1 | 0.204939 | 2002 |
| 8 | ARSLAN G | 529 | 1 | 1 | 1 | 0.038 | 1 | 0.194936 | 2000 |
| 9 | FREDHOLM BB | 529 | 1 | 1 | 1 | 0.038 | 1 | 0.194936 | 2000 |
| 10 | HALLDNER L | 529 | 1 | 1 | 1 | 0.038 | 1 | 0.194936 | 2000 |
| 11 | KULL B | 529 | 1 | 1 | 1 | 0.038 | 1 | 0.194936 | 2000 |
| 12 | SCHULTE G | 529 | 1 | 1 | 1 | 0.038 | 1 | 0.194936 | 2000 |
| 13 | WASSERMAN W | 529 | 1 | 1 | 1 | 0.038 | 1 | 0.194936 | 2000 |
| 14 | VON KUGELGEN I | 446 | 1 | 1 | 1 | 0.038 | 1 | 0.194936 | 2000 |
| 15 | WETTER A | 446 | 1 | 1 | 1 | 0.038 | 1 | 0.194936 | 2000 |
| 16 | FICHNA J | 439 | 2 | 2 | 2 | 0.133 | 2 | 0.515752 | 2011 |
| 17 | WIELAND T | 419 | 4 | 4 | 4 | 0.148 | 4 | 0.769415 | 1999 |
| 18 | FEELISCH M | 320 | 1 | 1 | 1 | 0.036 | 1 | 0.189737 | 1998 |
| 19 | FROMM MF | 318 | 1 | 1 | 1 | 0.05 | 1 | 0.223607 | 2006 |
| 20 | GRADHAND U | 318 | 1 | 1 | 1 | 0.05 | 1 | 0.223607 | 2006 |
| 21 | KÖNIG J | 318 | 1 | 1 | 1 | 0.05 | 1 | 0.223607 | 2006 |
| 22 | SEITHEL A | 318 | 1 | 1 | 1 | 0.05 | 1 | 0.223607 | 2006 |
| 23 | PIECHOTA-POLANCZYK A | 317 | 1 | 1 | 1 | 0.083 | 1 | 0.288097 | 2014 |
| 24 | KLOTZ K-N | 308 | 1 | 1 | 1 | 0.038 | 1 | 0.194936 | 2000 |
| 25 | INGELMAN-SUNDBERG M | 303 | 1 | 1 | 1 | 0.045 | 1 | 0.212132 | 2004 |
| 26 | TSUJIMOTO G | 267 | 1 | 1 | 1 | 0.059 | 1 | 0.242899 | 2009 |
| 27 | ILLES P | 249 | 2 | 2 | 2 | 0.077 | 2 | 0.392428 | 2000 |
| 28 | HOSSEINZADEH H | 248 | 3 | 3 | 3 | 0.3 | 3 | 0.948683 | 2016 |
| 29 | GEYER J | 237 | 2 | 2 | 2 | 0.1 | 2 | 0.447214 | 2006 |
| 30 | PETZINGER E | 237 | 2 | 2 | 2 | 0.1 | 2 | 0.447214 | 2006 |
| 31 | ALEMANY R | 217 | 1 | 1 | 1 | 0.053 | 1 | 0.230217 | 2007 |
| 32 | DANNEBERG K | 217 | 1 | 1 | 1 | 0.053 | 1 | 0.230217 | 2007 |
| 33 | MEYER ZU HERINGDORF D | 217 | 1 | 1 | 1 | 0.053 | 1 | 0.230217 | 2007 |
| 34 | TER BRAAK M | 217 | 1 | 1 | 1 | 0.053 | 1 | 0.230217 | 2007 |
| 35 | VAN KOPPEN CJ | 217 | 1 | 1 | 1 | 0.053 | 1 | 0.230217 | 2007 |
| 36 | KAUMANN AJ | 216 | 2 | 2 | 2 | 0.067 | 2 | 0.36606 | 1996 |
| 37 | MAYER B | 208 | 2 | 2 | 2 | 0.065 | 2 | 0.360555 | 1995 |
| 38 | BRUNS C | 205 | 1 | 1 | 1 | 0.031 | 1 | 0.176068 | 1994 |
| 39 | HOYER D | 205 | 1 | 1 | 1 | 0.031 | 1 | 0.176068 | 1994 |
| 40 | LÜBBERT H | 205 | 1 | 1 | 1 | 0.031 | 1 | 0.176068 | 1994 |
| 41 | NORENBERG W | 205 | 1 | 1 | 1 | 0.038 | 1 | 0.194936 | 2000 |
| 42 | CHATTERJEE PK | 188 | 1 | 1 | 1 | 0.053 | 1 | 0.230217 | 2007 |
| 43 | HAAS HL | 188 | 1 | 1 | 1 | 0.038 | 1 | 0.194936 | 2000 |
| 44 | KENNY B | 188 | 1 | 1 | 1 | 0.032 | 1 | 0.178885 | 1995 |
| 45 | SCHWINN DA | 188 | 1 | 1 | 1 | 0.032 | 1 | 0.178885 | 1995 |
| 46 | SELBACH O | 188 | 1 | 1 | 1 | 0.038 | 1 | 0.194936 | 2000 |
| 47 | BRODDE O-E | 180 | 2 | 2 | 2 | 0.091 | 2 | 0.426615 | 2004 |
| 48 | FILAROWSKA J | 174 | 1 | 1 | 1 | 0.143 | 1 | 0.378153 | 2019 |
| 48 | GAWEL K | 174 | 1 | 1 | 1 | 0.143 | 1 | 0.378153 | 2019 |
| 48 | GIBULA E | 174 | 1 | 1 | 1 | 0.143 | 1 | 0.378153 | 2019 |
| 48 | KOTLINSKA JH | 174 | 1 | 1 | 1 | 0.143 | 1 | 0.378153 | 2019 |
| 48 | MARSZALEK-GRABSKA M | 174 | 1 | 1 | 1 | 0.143 | 1 | 0.378153 | 2019 |
| 49 | MOLENAAR P | 172 | 1 | 1 | 1 | 0.034 | 1 | 0.184391 | 1997 |
| 50 | CENTURIÓN D | 166 | 1 | 1 | 1 | 0.053 | 1 | 0.230217 | 2007 |
| 50 | VILLALÓN CM | 166 | 1 | 1 | 1 | 0.053 | 1 | 0.230217 | 2007 |

**Supplementary Table 4:** The 50 most productive authors in the **top 100 most cited reviews** of *Naunyn-Schmiedeberg’s Archives of Pharmacology* (1969–2024), ranked based on total citations.
