# Supplementary material for: A bibliometric study of the most-cited research articles and reviews in Naunyn–Schmiedeberg’s Archives of Pharmacology (1969–2024)
Source: Naunyn Schmiedebergs Arch Pharmacol. 2025 Aug 1;399(1):1211–33. doi: 10.1007/s00210-025-04471-7 (PMC12894139; doi:10.1007/s00210-025-04471-7)
Supplement: Supplementary file 2 — (DOCX 696 KB) [file 210_2025_4471_MOESM2_ESM.docx]

**Legends**

**Supplementary Figure 1: The Top Ten Authors' Dynamics**. Publication trends of the ten most prolific authors in *Naunyn-Schmiedeberg’s Archives of Pharmacology* (1969–2024), showing variations in research output over time. **The data is for all research articles.**

**Supplementary Figure 2: The Top Universities' Dynamics.** Publication trends of the most productive universities in *Naunyn-Schmiedeberg’s Archives of Pharmacology* (1969–2024), highlighting institutional contributions over the years. **The data is for all research articles.**

**Supplementary Figure 3: The Top Ten Authors' Dynamics**. Publication trends of the ten most prolific authors in *Naunyn-Schmiedeberg’s Archives of Pharmacology* (1969–2024), showing variations in research output over time. **The data is for all reviews.**

**Supplementary Figure 4: The Top Universities' Dynamics.** Publication trends of the most productive universities in *Naunyn-Schmiedeberg’s Archives of Pharmacology* (1969–2024), highlighting institutional contributions over the years. **The data is for all reviews.**


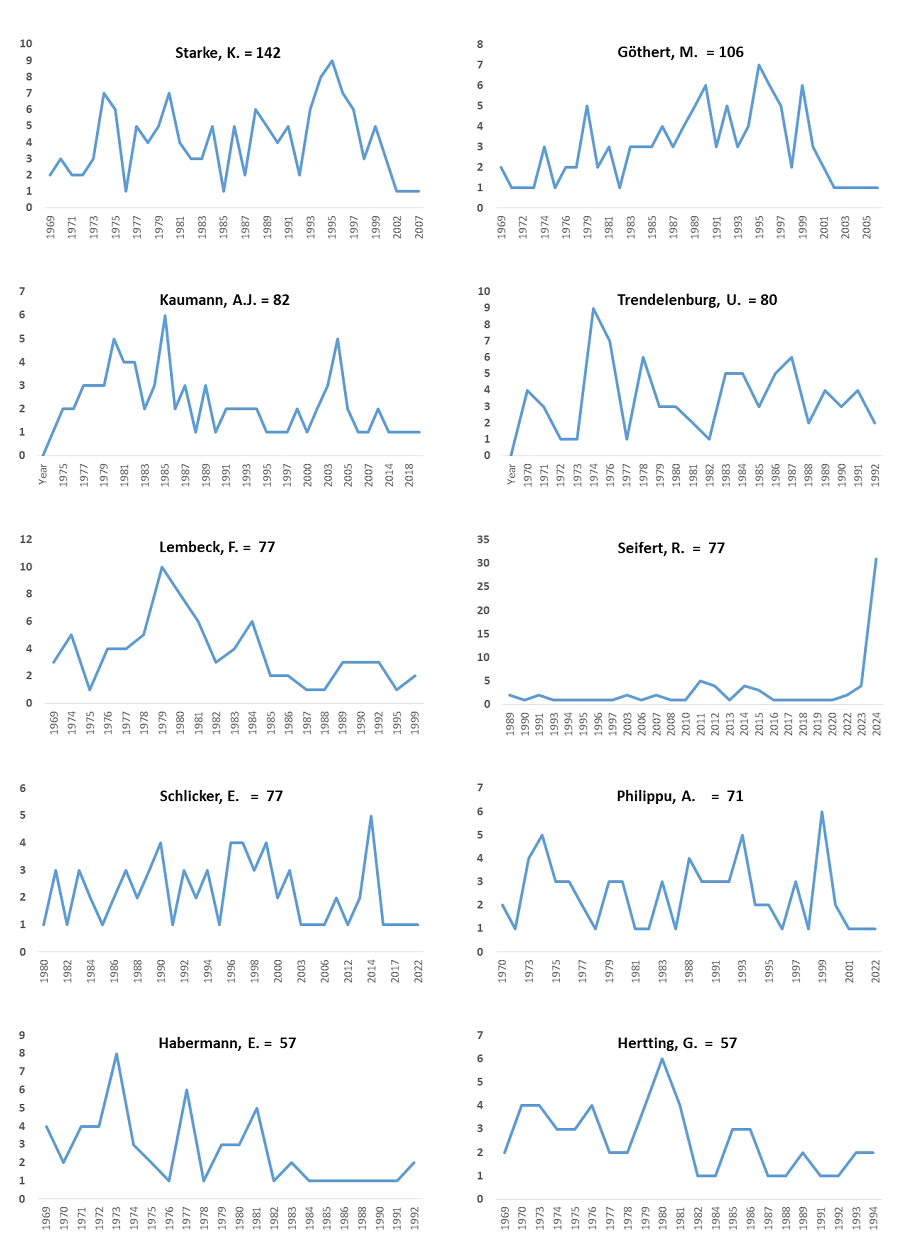


**Figure 1: The Top Ten Authors' Dynamics**. Publication trends of the ten most prolific authors in *Naunyn-Schmiedeberg’s Archives of Pharmacology* (1969–2024), showing variations in research output over time. **The data is for all research articles.**

| Ranking # | Name of University |
| --- | --- |
|  | 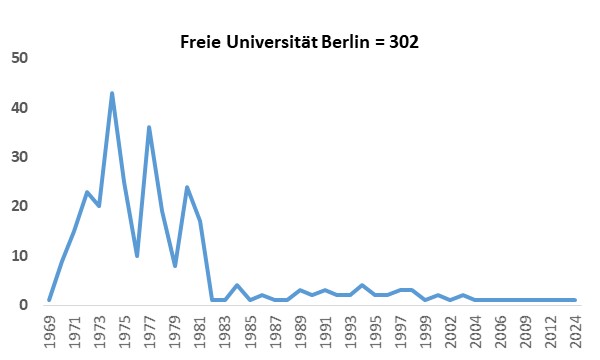 |
|  | 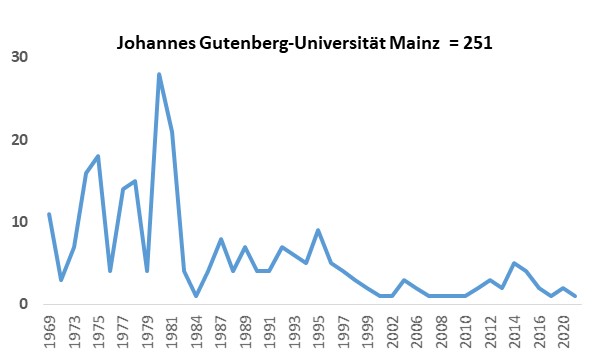 |
|  | 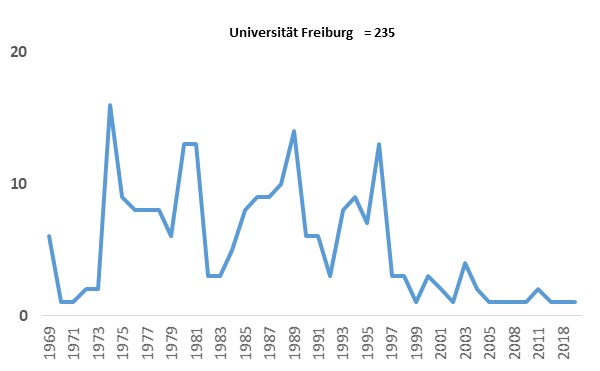 |
|  | 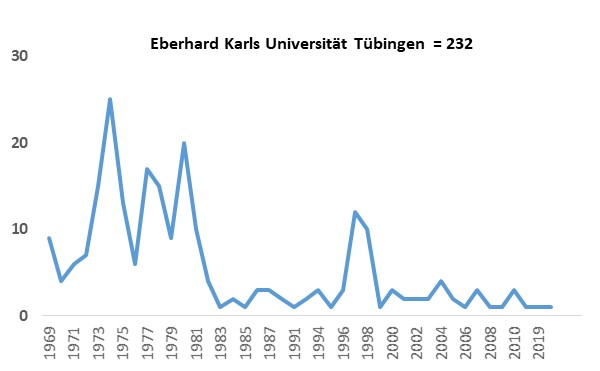 |
|  | 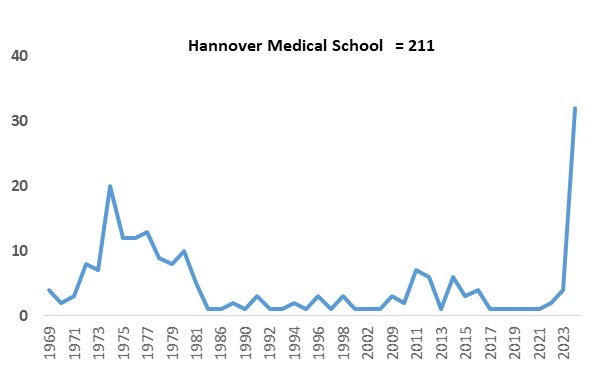 |
|  | 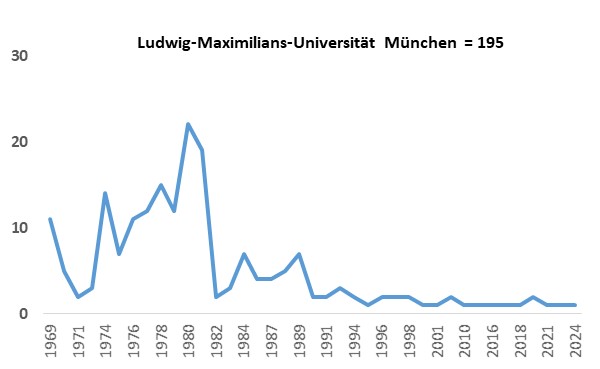 |
|  | 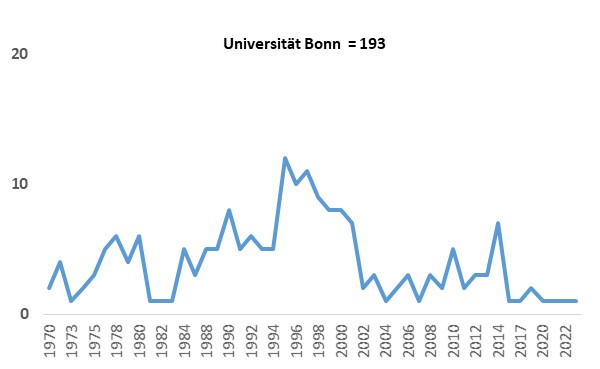 |
|  | 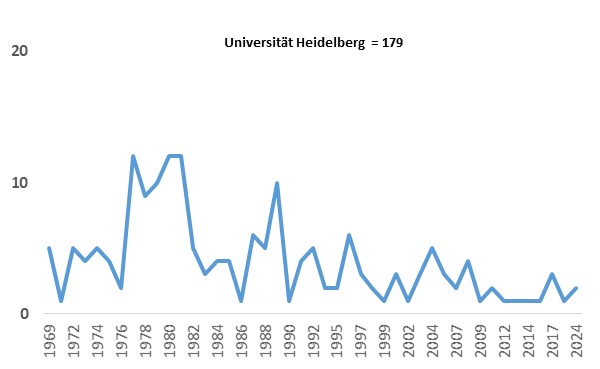 |
|  | 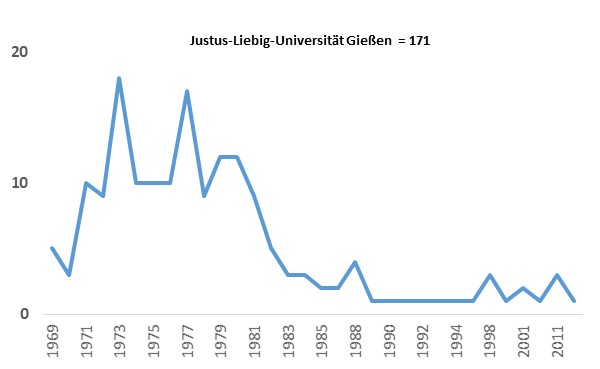 |
|  | 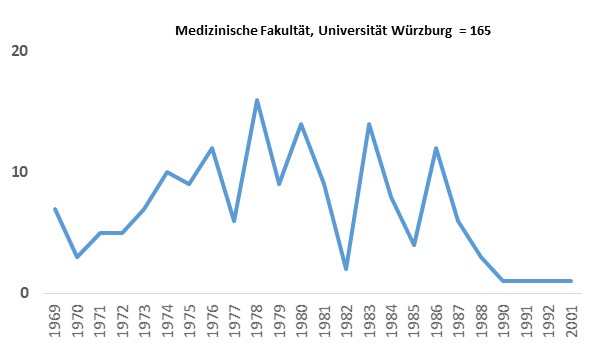 |

**Figure 2: The Top Universities' Dynamics.** Publication trends of the most productive universities in *Naunyn-Schmiedeberg’s Archives of Pharmacology* (1969–2024), highlighting institutional contributions over the years. **The data is for all research articles.**

| **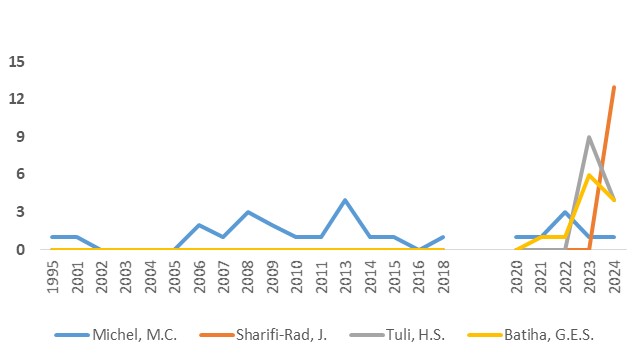** |
| --- |
| **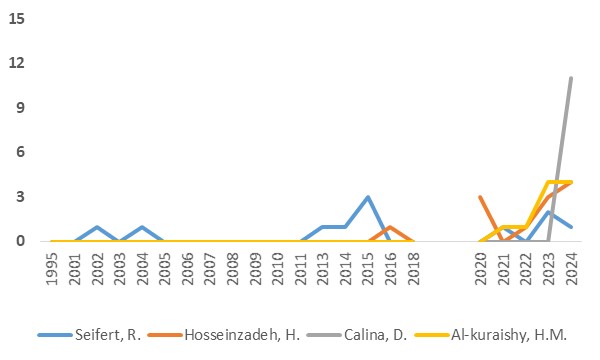** |

**Figure 3: The Top Ten Authors' Dynamics**. Publication trends of the ten most prolific authors in *Naunyn-Schmiedeberg’s Archives of Pharmacology* (1969–2024), showing variations in research output over time. **The data is for all reviews.**

| **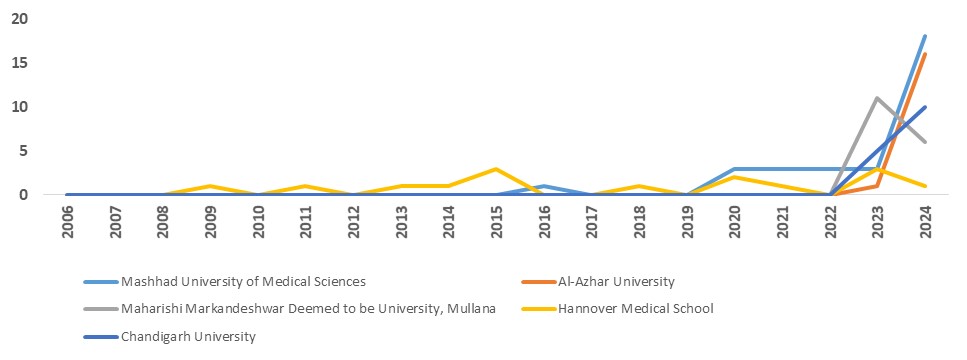** |
| --- |
| **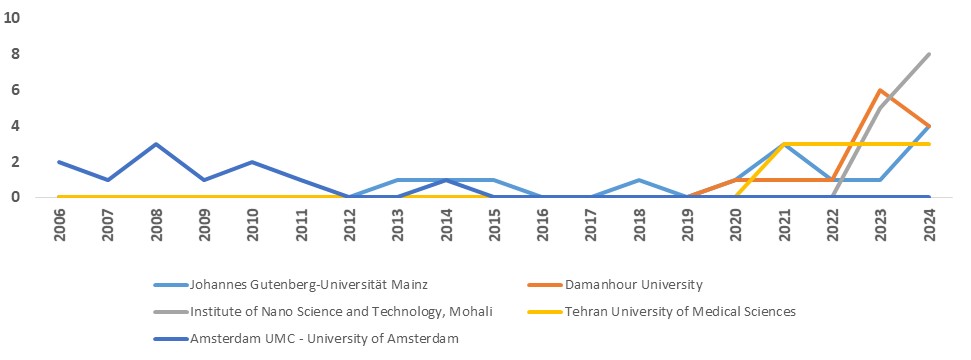** |

**Figure 4: The Top Universities' Dynamics.** Publication trends of the most productive universities in *Naunyn-Schmiedeberg’s Archives of Pharmacology* (1969–2024), highlighting institutional contributions over the years. **The data is for all reviews.**
